# Tumor–Immune Interactions in Pediatric Oral Rhabdomyosarcoma: A Narrative Review on Immuno-Oncology and Emerging Therapies

**DOI:** 10.3390/children12091249

**Published:** 2025-09-17

**Authors:** Omar A. El Meligy, Noha M. Elemam, Wael A. Hassan, Iman M. Talaat

**Affiliations:** 1 Pediatric Dentistry and Dental Public Health Department, Faculty of Dentistry, Alexandria University, Alexandria 21131, Egypt; omar.elmeligi@dent.alex.edu.eg; 2 Clinical Sciences Department, College of Medicine, University of Sharjah, Sharjah 27272, United Arab Emirates; nelemam@sharjah.ac.ae (N.M.E.); wael.hassan@sharjah.ac.ae (W.A.H.); 3 Research Institute of Medical & Health Sciences, University of Sharjah, Sharjah 27272, United Arab Emirates; 4 Department of Pathology, Faculty of Medicine, Suez Canal University, Ismailia 41522, Egypt; 5 Pathology Department, Faculty of Medicine, Alexandria University, Alexandria 21131, Egypt

**Keywords:** pediatric rhabdomyosarcoma, tumor immune microenvironment, immunotherapy, CAR therapies, immune checkpoints

## Abstract

Pediatric oral rhabdomyosarcoma (RMS) is a rare and aggressive cancer of the head and neck, characterized by a complex and mostly immunosuppressive tumor–immune microenvironment. Unlike adult cancers, pediatric RMS typically exhibits a “cold” immune profile, characterized by minimal T-cell infiltration, a low mutational burden, and resistance to immune checkpoint blockade. The tumor’s location in the oral cavity adds difficulty to treatment because of anatomical and functional limitations. Additionally, the presence of fusion oncogenes, such as PAX3:FOXO1, hampers immunogenicity and treatment response by disrupting antigen presentation and reducing immune cell infiltration. Advances in immuno-oncology have introduced new strategies, including immune checkpoint inhibitors, chimeric antigen receptor (CAR) therapies, cancer vaccines, and oncolytic viruses. However, these approaches face specific challenges in the pediatric population due to developmental immune factors. This narrative review highlights recent findings on the immunobiology of pediatric oral RMS, focusing on tumor–immune interactions and their impact on disease progression and treatment resistance. We reviewed the cellular components of the TIME, the mechanisms of immune evasion, and the expression of immune checkpoints, including PD-L1 and B7-H3. Emerging immunotherapies, including CAR-T, CAR-NK, and CAR-CIK cell therapies; checkpoint inhibitors; oncolytic viruses; and cancer vaccines, are discussed, with an emphasis on their current limitations and potential to transform the pediatric RMS immune landscape.

## 1. Introduction

Rhabdomyosarcoma (RMS) is the most common soft tissue sarcoma in children, frequently arising in the oral cavity as well as the head and neck regions. Oral RMS, often linked to the embryonal subtype, presents unique therapeutic challenges due to its anatomical location and potential impact on craniofacial development. Immune surveillance is critical for recognizing and eliminating tumor cells, and the tumor–immune microenvironment (TIME) in pediatric oral RMS has become an area of growing research interest. Compared to adult tumors, pediatric tumors exhibit distinct features in immune cell infiltration, antigen presentation, and stromal interactions. Many pediatric RMS tumors exhibit a “cold” immune profile, characterized by low expression of immune checkpoints and limited T-cell infiltration [1], underscoring the need for strategies that elicit robust anti-tumor responses. Recent advances in immuno-oncology offer promising therapeutic avenues for pediatric RMS, including immune checkpoint inhibitors, chimeric antigen receptor (CAR) T and NK cell therapies, cancer vaccines, and oncolytic viruses. Preclinical and early clinical studies indicate that these approaches can remodel the TIME, enhance T-cell activation, and mitigate tumor-induced immunosuppression [2,3]. Nevertheless, their safety and efficacy in children, particularly in oral tumors, remain under active investigation. This review provides a comprehensive exploration of tumor–immune interactions in pediatric oral RMS, with a focus on the TIME and implications for immunotherapy. Our objectives are to: (1) characterize the immunological landscape of pediatric oral RMS, highlighting key cellular and molecular components of the TIME; (2) identify mechanisms of immune evasion and potential therapeutic targets; and (3) critically evaluate the clinical relevance and limitations of emerging immunotherapies, including CAR-T cells, checkpoint inhibitors, and cancer vaccines. By integrating current published evidence, this narrative review aims to present a coherent perspective on tumor–immune dynamics in pediatric oral RMS.

### 1.1. Overview of Pediatric Rhabdomyosarcoma

RMS is the most common soft tissue sarcoma in the pediatric population, accounting for approximately 5% of all childhood malignancies and nearly 50% of pediatric soft tissue sarcomas [4]. It arises from primitive mesenchymal progenitor cells committed to skeletal muscle differentiation and displays notable histological and clinical heterogeneity [5]. The estimated annual incidence is 4.6 cases per million individuals under 20 years of age, with around 350 new diagnoses occurring each year in the United States [6].

RMS is histologically categorized into four primary subtypes: embryonal (ERMS), alveolar (ARMS), spindle cell/sclerosing, and pleomorphic. ERMS is the most common form, representing 60–70% of cases, typically affecting younger children and commonly presenting in the head and neck region [5,7]. This subtype generally has a more favorable prognosis. In contrast, ARMS more frequently affects older children and adolescents, is clinically more aggressive, and is strongly associated with fusion oncogenes such as PAX3:FOXO1 and PAX7:FOXO1. These genetic alterations serve as key biomarkers, possessing diagnostic, prognostic, and biological significance, which influence tumor behavior and immune evasion mechanisms [8,9].

RMS can occur in various anatomical locations, with the head and neck (28%), extremities (24%), and genitourinary tract (18%) being the most commonly affected areas [6,10]. Multimodal treatment, which includes surgery, chemotherapy, and radiotherapy, is the standard approach and is tailored to the specific histological subtype, tumor location, and risk category. Although outcomes for localized RMS have improved significantly due to advances in therapy, prognosis remains poor for patients with metastatic, recurrent, or fusion-positive disease. In these high-risk groups, the five-year overall survival rate often remains below 30% [9,11].

### 1.2. Epidemiology and Clinical Significance of Oral Rhabdomyosarcoma

Oral RMS, although relatively rare, represents a clinically important subset of head and neck RMS due to the complex anatomy and vital functional roles of the oral cavity. The head and neck region accounts for approximately 28–35% of pediatric RMS cases, with 10–15% specifically involving the oral cavity and oropharyngeal structures such as the tongue, palate, buccal mucosa, gingiva, and floor of the mouth [12].

Tumors in this area often present with nonspecific symptoms, such as localized swelling, discomfort, ulceration, or impaired function, that may resemble benign conditions like infections or reactive lesions. This often causes delays in diagnosis and treatment, leading to advanced-stage disease at the time of presentation [13]. Due to the complex anatomy of the oral cavity and its role in vital activities like speech, swallowing, and chewing, it is frequently impossible to achieve complete surgical removal with clear margins without risking significant functional loss [14].

Therefore, treatment approaches usually focus on systemic chemotherapy and radiotherapy rather than radical surgery. However, these treatments carry significant short- and long-term side effects. In pediatric patients, radiotherapy and cytotoxic drugs can cause growth issues, craniofacial asymmetry, dental problems, soft tissue fibrosis, and long-term neurocognitive impairments [15]. Moreover, the risk of local recurrence remains high, and the overall complications related to oral RMS are substantial. These issues highlight the importance of precision oncology and immunotherapy strategies that aim to preserve quality of life while achieving effective cancer control (Table 1).

### 1.3. Importance of the Tumor Immune Microenvironment in Pediatric Rhabdomyosarcoma

TIME plays a crucial role in cancer initiation, progression, metastasis, and treatment response. Although pediatric tumors, including RMS, have traditionally been viewed as less immunologically complex than adult cancers, new insights are challenging this notion. Pediatric RMS, especially the alveolar subtype with PAX3:FOXO1 or PAX7:FOXO1 fusions, often exhibits a notably immune-suppressing environment that impairs effective anti-tumor immune responses [16,17].

RMS tumors typically exhibit an “immune-cold” phenotype, characterized by a low tumor mutational burden (TMB), decreased neoantigen presentation, and limited infiltration of effector immune cells, such as cytotoxic CD8^+^ T lymphocytes [18]. Instead, these tumors are often rich in immunosuppressive cell populations, including tumor-associated macrophages (TAMs), regulatory T cells (Tregs), and myeloid-derived suppressor cells (MDSCs), which collectively drive immune evasion and tumor growth [19].

Additionally, oncogenic fusion genes such as PAX3:FOXO1 have been implicated in suppressing interferon signaling pathways and reducing the expression of antigen presentation machinery, further impairing immune recognition of tumor cells [20]. Single-cell transcriptomic analyses have highlighted the heterogeneity of immune cell infiltration in pediatric sarcomas, revealing immune-suppressive niches even in tumors with minimal overall immune cell presence [21].

These findings highlight the significance of TIME in RMS pathobiology and therapeutic resistance. They also show the potential of immunomodulatory strategies to reprogram the tumor microenvironment (TME), enhance immune infiltration, and make tumors more responsive to immunotherapy, thereby drawing more attention to the role of TIME in pediatric oncology research and drug development.

### 1.4. Need for Immuno-Oncology Approaches in Rhabdomyosarcoma

Despite notable advances in multimodal treatment strategies, survival outcomes for high-risk, metastatic, or recurrent pediatric RMS remain inadequate. Traditional cytotoxic regimens, such as chemotherapy and radiotherapy, have plateaued in effectiveness, emphasizing the urgent need for new therapeutic approaches. Immuno-oncology, which utilizes the body’s immune system to target and destroy tumor cells, presents a promising strategy for addressing these challenges in RMS [22].

While immune checkpoint inhibitors (ICIs), such as anti-PD-1/PD-L1 and anti-CTLA-4 antibodies, have revolutionized cancer treatment in adults, their application in pediatric sarcomas has shown limited success. This is primarily due to the intrinsic immunogenic properties of pediatric tumors and the immunosuppressive nature of their microenvironment [23]. However, recent developments have spurred interest in alternative immune-oncology strategies tailored for RMS, including adoptive T cell therapy, cancer vaccines, and macrophage reprogramming [24].

For instance, preclinical studies have demonstrated that chimeric antigen receptor T (CAR-T) cells targeting receptor molecules such as FGFR4 and HER2 can trigger cytotoxic immune responses in RMS models, suggesting their potential for clinical application [1]. In addition, combinatorial approaches are being explored to enhance the efficacy of ICIs. These strategies involve pairing ICIs with agents that modulate the TME, such as colony-stimulating factor-1 receptor (CSF1R) inhibitors, to transform “immune-cold” RMS tumors into “hot” phenotypes that are more susceptible to immune system attack [25].

Immunotherapeutic approaches currently under investigation for RMS include: immune checkpoint inhibitors targeting the PD-1/PD-L1 and CTLA-4 pathways, CAR-T cell therapy, particularly against tumor antigens such as HER2 and B7-H3, oncolytic virotherapy, which uses engineered viruses to selectively infect and kill tumor cells while stimulating systemic immune responses, tumor-infiltrating lymphocyte (TIL) therapy, aimed at enhancing local immune surveillance and cytotoxic activity, and cancer vaccines designed to elicit immune responses specific to tumor-associated antigens [26,27,28].

Although early-phase clinical trials have shown limited effectiveness, especially in fusion-positive RMS, combining immunotherapy with traditional treatments and epigenetic reprogramming shows promise. Gaining a deeper understanding of TIME and its interactions with tumor biology is essential for developing successful immunotherapeutic strategies. Personalized therapies that consider the immune environment could provide new hope for improving outcomes in pediatric RMS, particularly for those with metastatic or recurrent disease [29,30].

Therefore, by integrating perspectives from immunology, oncology, and pediatric pathology, this review aims to create a framework for advancing immuno-oncology strategies in pediatric RMS. The ultimate goal is to improve clinical outcomes for children affected by this aggressive and often difficult-to-treat cancer.

## 2. Tumor–Immune Microenvironment in Oral Rhabdomyosarcoma

The TME encompasses the complex surroundings surrounding tumor cells, including immune cells, stromal cells, blood vessels, and signaling molecules [31]. These components work together to form a unique environment that can either hinder or encourage tumor growth and spread [32]. The TIME features a diverse range of immune cell types within the TME, including tumor-infiltrating lymphocytes, Tregs, natural killer (NK) cells, and macrophages [33]. In oral RMS, each of these cell types has a specific role in shaping the tumor’s immune profile, and the interactions among these cells within the TIME affect not only how the tumor progresses but also how well treatments work [13].

### 2.1. Tumor-Infiltrating Immune Cells in Oral Rhabdomyosarcoma

#### 2.1.1. Macrophages

Tumor-associated macrophages (TAMs) are prominent components of the TIME, and their polarization state (M1 or M2) determines their function: M1 Macrophages are classically activated macrophages that are induced by stimuli such as lipopolysaccharides (LPS) and interferon-gamma (IFN-γ), and are characterized by the production of pro-inflammatory cytokines and promotion of Th1 responses [34]. M2 macrophages, on the other hand, are alternatively activated macrophages induced by cytokines such as IL-4, IL-10, and TGF-β. They secrete anti-inflammatory cytokines that contribute to tumor growth, angiogenesis, and immune suppression [34]. Such polarization is regulated by various signaling pathways, including JAK-STAT, MAPK, and PI3K-AKT pathways, which mediate the effects of these cytokines on macrophage differentiation [35].

In oral RMS, single-cell transcriptomic analysis revealed that macrophages predominantly exhibited an M2 polarization state [36]. In addition, Rutland et al. (2023) have identified the presence of Schwann cells within the RMS microenvironment, yet their functional significance and mechanistic contributions to tumor biology remain poorly characterized [37]. Recently, it has been demonstrated that Schwann cells within the RMS secrete macrophage migration inhibitory factor (MIF) and pleiotrophin (PTN), which interact with their respective receptors on macrophages (CD74 and SDC3), thereby promoting their polarization towards the M2 phenotype [38], ultimately leading to immunosuppression and tumor progression.

#### 2.1.2. Lymphocytes

T lymphocytes, including CD4^+^ T helper (Th) cells, CD8^+^ cytotoxic T cells (CTLs), and Tregs, play distinct roles in the immune response. CD4^+^ T cells are activated by tumor antigens presented by antigen-presenting cells (APCs) and produce cytokines such as IFN-γ and tumor necrosis factor-alpha (TNF-α), which in turn activate CD8^+^ CTLs and macrophages [39]. CD8^+^ T cells recognize and kill tumor cells presenting specific antigens via MHC class I molecules [39]. This interaction between CD4^+^ and CD8^+^ T cells is crucial for an effective anti-tumor immune response. Tregs, characterized by the expression of FOXP3, have a dual role in the TME. They can suppress immune responses by inhibiting the activity of CD4^+^ and CD8^+^ T cells, thus promoting tumor immune escape [40]. Conversely, they can support a balanced immune system that may be essential in preventing tumor metastasis and recurrence [41].

In oral RMS, studies have shown that high infiltration of CD4^+^ and CD8^+^ T cells into the tumor is associated with a better prognosis and improved patient outcomes [42,43]. On the other hand, high levels of Tregs in the blood and tumor tissue of patients have been correlated with poor clinical outcomes [44]. Thus, an effective anti-tumor response requires robust activation of CD4^+^ and CD8^+^ T cells, while excessive Treg activity can dampen this response and promote tumor progression.

##### T Cell Exhaustion and Dysfunction in the Oral Rhabdomyosarcoma Microenvironment

Studies have shown that chronic exposure to tumor-associated antigens within the tumor TME leads to a progressive loss of T cell effector functions, such as T cell exhaustion [43]. This state is characterized by the sustained upregulation of inhibitory receptors: programmed cell death protein 1 (PD-1) and cytotoxic T-lymphocyte-associated protein 4 (CTLA-4) [41]. Exhausted T cells display diminished cytokine production, impaired proliferation, and reduced cytotoxicity, ultimately weakening anti-tumor immunity and correlating with poor clinical outcomes [42,44]. Recent studies have demonstrated that the expression of Fibroblast Growth Factor Receptor 4 (FGFR4) and CD276 (also known as B7-H3) on tumor cells and within the TME contributes to the suppression of anti-tumor immune responses, especially by inhibiting the activity of both CD4^+^ and CD8^+^ T lymphocytes through T cell exhaustion [45]. Therefore, chimeric antigen receptor (CAR) T cells have been engineered to target FGFR4 and CD276, thereby successfully reducing T cell exhaustion, enhancing T cell persistence, and restoring a robust anti-tumor immune response [45]. This strategy represents a promising advancement in immunotherapy for the treatment of RMS.

Moreover, recent research has highlighted the role of NECTIN3-TIGIT interactions in the immune evasion mechanisms of fusion-positive RMS (characterized by PAX3-FOXO1 or PAX7-FOXO1 gene fusions). NECTIN3, an immune checkpoint ligand expressed on RMS cells, can engage the TIGIT receptor, which is broadly expressed on both CD4^+^ and CD8^+^ T cells within the TME of RMS. The engagement of TIGIT by NECTIN3 on tumor cells results in the functional impairment of both T cell subsets, thereby reducing their ability to mount an effective anti-tumor response and promoting tumor immune evasion. This interaction has been associated with poorer patient outcomes, as it allows the tumor to escape immune surveillance [36].

##### Regulatory T Cell (Treg) Plasticity and Tumor Promotion in Oral Rhabdomyosarcoma

Tregs demonstrate plasticity in the RMS microenvironment, where they can be “educated” by the TME, leading to their reprogramming into immune-suppressive roles that support tumor growth and metastasis [46]. This education occurs through several mechanisms: the presence of TAMs of the M2 polarized subtype within the TME secretes immunosuppressive cytokines such as TGF-β and IL-10, which promote the expansion and functional adaptation of Tregs [47]. Additionally, metabolic changes in the TME, such as increased lactate production by tumor cells, further polarize macrophages to the M2 subtype and support Treg expansion. Moreover, the ectopic expression of CD137 on RMS cells, which downregulates CD137L on APCs, thus reduces T cell co-stimulation and diminishes T cell-mediated killing of RMS cells [48]. Furthermore, Tregs in the TME can upregulate genes associated with tissue repair and angiogenesis, further promoting tumor progression [41]. The net effect is a feedback loop where RMS cells and their microenvironment continually reinforce Treg-mediated immunosuppression.

##### CD4^+^ CTLs in Oral Rhabdomyosarcoma

CD4^+^ CTLs are a subset of T cells with cytotoxic activity, characterized by the expression of perforin and granzyme B, enabling them to kill infected or malignant cells, similar to CD8^+^ CTLs [49]. In the context of RMS, the TME is enriched with CD4^+^ CTLs that can be stimulated by fusion peptides derived from the PAX-FKHR fusion protein in RMS cells, leading to the lysis of tumor cells [50]. Therefore, their presence in the TME has been linked to better prognoses in RMS patients [51]. Interestingly, in oral squamous cell carcinoma, expansion of these CD4^+^ CTLs has been associated with immune suppression, especially following interventions such as chimeric antigen receptor (CAR)-T cell therapy [52]. This suggests a potential role for these cells in promoting tumor immune evasion, possibly through the upregulation of inhibitory receptors, such as PD-1, and the modulation of the local immune microenvironment [52]. Although direct evidence for these mechanisms in RMS is limited, similar immunosuppressive pathways seen in oral squamous cell carcinoma offer a basis for further study in RMS.

#### 2.1.3. Myeloid-Derived Suppressor Cells

MDSCs constitute a heterogeneous population of immature myeloid cells characterized by their immunosuppressive capabilities. In cancer patients, the accumulation of MDSCs is observed in the peripheral blood, draining lymphoid tissues, and tumor sites [53], where they form immunosuppressive networks. These cells exhibit significant proliferation and expansion with neoplastic progression, as they facilitate tumor immune evasion, angiogenesis, and tumor invasion [53]. MDSCs are broadly categorized into polymorphonuclear (PMN-MDSCs) and monocytic (M-MDSCs) subsets, both of which utilize mechanisms such as arginase-1 (ARG1), reactive oxygen species (ROS), and nitric oxide (NO) to inhibit CTLs and NK cells [54]. Furthermore, experimental and clinical evidence demonstrate that MDSCs significantly attenuate the efficiency of current anti-tumor strategies such as chemotherapy, radiotherapy, and immunotherapy [55].

##### Recruitment of MDSCs in the Rhabdomyosarcoma Microenvironment

Several cytokines in the TME have been identified as major drivers of MDSC expansion, especially Signal Transducer and Activator of Transcription 3 (STAT3), which enhances MDSC proliferation and survival through STAT3 activation [53]. Colony-stimulating factors, including granulocyte-macrophage colony-stimulating factor (GM-CSF), macrophage colony-stimulating factor (M-CSF), and granulocyte colony-stimulating factor (G-CSF), are crucial for regulating myeloid cell differentiation and MDSC expansion. In a syngeneic orthotopic RMS model that metastasizes to the lung, MDSCs were the dominant immune cells recruited to pre-metastatic lung sites [17]. Additionally, preclinical studies demonstrate the accumulation of CXCR2^+^ PMN-MDSCs in the RMS microenvironment and metastatic niches, indicating that these cells are recruited to the TME via chemokines such as CCL2 and CXCL12, where they interact with tumor cells to maintain immunosuppression [56]. Disrupting CXCR2-mediated migration in MDSCs has been shown to significantly enhance the effectiveness of anti-PD1 treatments, suggesting that CXCR2 could serve as a target to prevent MDSC recruitment and improve immunotherapeutic interventions [57].

##### Activation of MDSCs in Rhabdomyosarcoma

The TME in RMS is influenced by metabolic factors such as adenosine, which has been shown to enhance the activity of MDSCs, making them more effective at blunting anti-tumor immunity and contributing to chemoresistance [58]. Moreover, in preclinical RMS models, tumor-derived G-CSF was identified as one of the key factors driving the expansion of MDSCs, thereby limiting the efficacy of chimeric antigen receptor (CAR) T-cell therapy [59]. Although not yet proved in RMS, studies in various other sarcomas and carcinomas have shown that tumor-derived exosomes (vesicles released by cancer cells) containing specific microRNAs (miRNAs), such as miR-10a and miR-21, that target several pathways in MDSCs, like NF-κB and PTEN/PI3K/AKT, which target genes involved in MDSCs, such as RORA and PTEN, thereby enhancing the immunosuppressive capacity of MDSCs within the TME [60]. Additionally, exosomes from MDSCs themselves can promote tumor cell proliferation and metastasis by conveying miRNAs such as miR-126a and miR-143-3p, which further contribute to the immunosuppressive environment and tumor progression [60].

##### Immunosuppressive Mechanisms of MDSCs in Rhabdomyosarcoma

The functional role of MDSCs in RMS includes suppressing CTLs and contributing to resistance against immunotherapy. These cells use various methods to inhibit anti-tumor immune responses, such as producing reactive oxygen species (ROS) and nitric oxide (NO), which induce oxidative stress and impair T cell function [61]. Additionally, MDSCs catalyze the nitration of T-cell receptor (TCR)/CD8 molecules, blocking the TCR/major histocompatibility complex-peptide interactions necessary for effective T cell activation [61]. They also produce ARG1, which depletes L-arginine from the microenvironment, a vital amino acid for T cell proliferation and activity [61]. Furthermore, MDSCs can suppress B-cell differentiation and function, mainly through mechanisms involving TGF-β-mediated Interleukin-7 (IL-7) deficiency, which diminishes downstream STAT-5 signaling required for B-cell development [62]. MDSCs also activate Tregs [61], creating an immunosuppressive environment that may facilitate RMS progression by enabling tumor cells to evade immune detection. These findings align with the immunophenotypic analysis by Nomikos et al. (2024), which examined the embryonal RMS TME and identified a predominant population of CD11b^+^ myeloid cells, commonly associated with MDSCs. Furthermore, quantification revealed significantly lower numbers of CD3^+^ T lymphocytes and B220^+^ B lymphocytes compared to the CD11b^+^ cells [57]. This unequal distribution of immune cells suggests an immune-suppressing environment within RMS tumors, likely dominated by MDSCs.

### 2.2. Immunosuppressive Cytokines and Chemokines in Oral Rhabdomyosarcoma

RMS employs multiple sophisticated mechanisms to evade immune surveillance, utilizing immunosuppressive cytokines, such as interleukin-10 (IL-10) and transforming growth factor-beta (TGF-β), as well as chemokines, including CCL2 and CXCL12, which play key roles.

#### 2.2.1. IL-10-Driven Immune Suppression in Rhabdomyosarcoma

IL-10, produced by both tumor cells and tumor-infiltrating immune cells, primarily acts by suppressing the activation and function of APCs and promoting the differentiation of Tregs [63,64]. Mechanistically, IL-10 inhibits the expression of MHC class II and costimulatory molecules on dendritic cells and macrophages, thereby reducing their ability to activate CD8^+^ T cells [65]. This suppression is further enhanced by IL-10’s induction of Tregs, which release additional immunosuppressive cytokines, such as IL-10 and TGF-β, thereby forming a feedback loop that maintains immune tolerance [66].

In RMS, a study by Kather et al. (2019) shows that CD163-positive macrophages, a subset characterized by a propensity for IL-10 production, are preferentially enriched within embryonal RMS and correlate with poorer survival outcomes [47]. These observations suggest that TAMs are a primary source of IL-10 within the RMS microenvironment. Collectively, these findings support the role of IL-10 as both a prognostic biomarker and a potential therapeutic target in RMS.

#### 2.2.2. Contextual “Jekyll and Hyde” of TGF-β Signaling in Rhabdomyosarcoma

TGF-β signaling in RMS exemplifies the contextual duality of cytokine activity. In early tumor stages, TGF-β acts as a tumor suppressor by inducing apoptosis and cell cycle arrest. However, in advanced RMS, TGF-β shifts to promote immune evasion via SMAD2/3-dependent pathways that drive Treg differentiation and inhibit CTLs [67].

Mesenchymal-like tumor cells and fibroblasts in the RMS microenvironment are the source of abundant TGF-β in this sarcoma [31] The transition of TGF-β signaling in RMS from a tumor-suppressive to tumor-promoting role is influenced by interactions between RMS cells and the surrounding stromal cells. Interestingly, cancer cells have the remarkable ability to “educate” nearby mesenchymal cells, gradually reprogramming TGF-β signaling to favor tumor growth. In a recent study by Zhang et al. (2025), the authors delved into this fascinating crosstalk. They explored tumor growth-regulatory signaling between differentially educated non-malignant mesenchymal stromal cells and malignant cells in pediatric RMS [64]. They found that TGFβ-inducible genes, specifically CTGF (also known as CCN2) and PAI-1 (also known as SERPINE1), were among the top downregulated genes in RMS cells when exposed to tumor growth-suppressive stromal cells. This suggests that the anti-TME can suppress the expression of these genes, which are typically upregulated by TGFβ signaling. The authors also report that the presence of CTGF increases RMS cell survival after three days in culture under starvation conditions, which indicates that CTGF can act as a survival factor for RMS cells, especially when nutrients are limited. Additionally, when the researchers compared gene expression in tumors with high versus low CTGF expression across two independent RMS patient cohorts, they found that SERPINE1 (PAI-1) was one of the top candidate genes co-regulated with CTGF [64]. This co-expression pattern suggests that TGFβ-inducible genes CTGF and SERPINE1 are regulated together in RMS, likely reflecting shared signaling pathways or regulatory mechanisms. All these observations highlight how the TME and the ongoing dialogue between cancer and stromal cells can reshape key signaling networks, ultimately influencing the fate of RMS tumors.

TGF-β exerts profound immunosuppressive effects within TME. One of its primary actions is the inhibition of NK cell cytotoxicity, thereby impairing a critical component of innate anti-tumor immunity [61]. TGF-β also suppresses the production of Th1 cytokines, which are essential for mounting an effective anti-tumor immune response through the activation of CTLs and the enhancement of NK cell activity [68]. In addition, TGF-β promotes the induction of regulatory B cells (Bregs) that secrete IL-10, further contributing to the immunosuppressive milieu [69]. TGF-β also plays a pivotal role in the differentiation of MDSCs and skewing macrophage polarization toward the tumor-promoting M2 phenotype [70]. Notably, elevated TGF-β levels have been associated with poor prognosis in patients with RMS, underscoring its significance as a potential therapeutic target [69].

#### 2.2.3. Chemokine-Mediated Immune Suppression in Rhabdomyosarcoma

The recruitment of MDSCs and TAMs in the TME of RMS is significantly influenced by chemokines such as CCL2 and CXCL12. CCL2, produced by RMS cells and stromal fibroblasts, binds to CCR2 on MDSCs, facilitating their infiltration and subsequent suppression of T-cell proliferation via ARG1 and ROS [65]. Similarly, CXCL12, via its receptor CXCR4, not only attracts PMN-MDSCs but also traps effector T cells in peritumoral stroma, limiting their access to cancer cells [38]. These chemokine networks are amplified by tumor-derived exosomes, which carry immunosuppressive ligands like PD-L1 and TGF-β, reprogramming distant immune cells to support metastasis [69]. Targeting these chemokine pathways could provide therapeutic strategies to block the recruitment of immunosuppressive cells, potentially enhancing anti-tumor immune responses in RMS.

### 2.3. Immune Evasion Mechanisms in Rhabdomyosarcoma

Recent advances have significantly deepened our understanding of the immunobiology of pediatric RMS, revealing a complex interplay between tumor cells and the immune microenvironment. Notably, emerging evidence suggests that RMS cells dynamically regulate immune checkpoint molecules, with programmed death-ligand 1 (PD-L1) expression being significantly upregulated in response to pro-inflammatory cytokines, such as IFN-γ.

While early studies using the SP142 clone detected negligible PD-L1 expression in RMS cells, newer data using the 22C3 antibody demonstrate PD-L1 positivity in 35% of cases, restricted to tumor-associated immune cells, which correlates with improved relapse-free survival in low-stage disease [70]. This adaptive PD-L1 upregulation, in concert with immunosuppressive cytokines IL-10 and TGF-β, orchestrates a profound T-cell exhaustion phenotype and functional impairment within the TME, thereby facilitating immune escape and tumor persistence.

RMS cells exhibit a near absence of major histocompatibility complex class I (MHC-I) expression, a critical determinant for CD8^+^ CTL recognition and tumor cell lysis. Intriguingly, this loss of antigen presentation is not attributable to genetic defects in the antigen processing and presentation machinery, but rather reflects a reversible, developmentally regulated epigenetic silencing reminiscent of early myogenic differentiation [69]. Recent studies have demonstrated that epigenetic modulators, such as DNA demethylating agents and histone deacetylase inhibitors, are capable of reactivating MHC-I surface expression on tumor cells [71]. This re-expression enhances the susceptibility of malignant cells to immune-mediated eradication, thereby potentiating the therapeutic impact of immunotherapeutic interventions. Furthermore, the anti-tumor properties of IL-15, attributed to its capacity to support the survival, proliferation, and effector functions of NK cells and CD8^+^ CTLs, have been substantiated in both preclinical models and clinical trials involving patients with RMS [68].

Beyond the well-characterized role of cytokines in immune modulation, RMS cells leverage a sophisticated array of surface molecules to escape immune surveillance. Among these, B7-H3 (CD276) has emerged as a particularly intriguing player. Strikingly, B7-H3 is overexpressed in approximately 70–80% of RMS tumors, while its expression is virtually undetectable in healthy skeletal muscle tissue [72]. This stark contrast not only underscores its potential as a highly selective therapeutic target but also raises important questions about its role in tumor biology. Mechanistically, B7-H3 acts as a potent immune checkpoint molecule. It suppresses the activation of CTLs and facilitates the recruitment of M2-polarized macrophages. This dual action contributes to the formation of an immune-excluded TME, effectively shielding RMS cells from immune attack [72].

Given these findings, B7-H3 is not only a promising target for novel immunotherapeutic strategies, such as monoclonal antibodies and CAR-T cell therapies, but also a potential indicator of tumor immune status. Ongoing clinical trials are now exploring B7-H3-targeted agents in pediatric RMS [73], to transform the treatment landscape for these challenging malignancies.

Key Remark: Pediatric oral RMS is characterized by a profoundly immunosuppressive microenvironment dominated by M2 macrophages, Tregs, and MDSCs. These immune cells collaborate with tumor-derived factors to inhibit the activity of cytotoxic T cells and promote immune escape. Understanding and targeting these components is critical for enhancing immunotherapy responsiveness.

**Figure 1:**
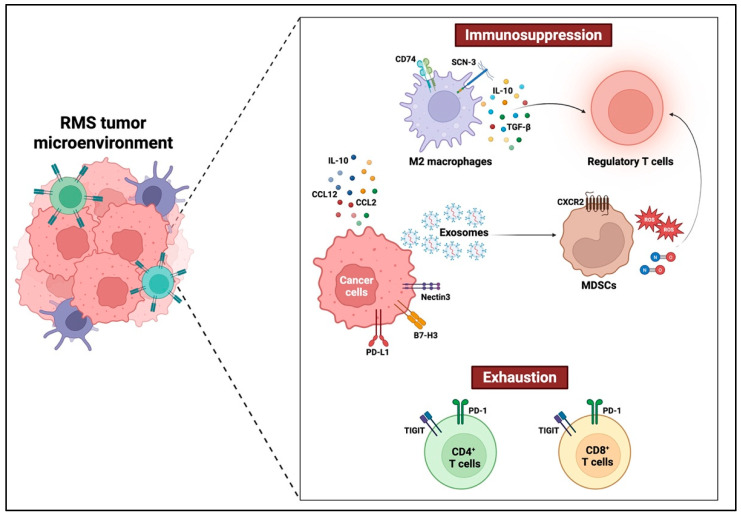
Immunosuppressive Features of the Tumor Immune Microenvironment in Pediatric Oral Rhabdomyosarcoma.

To facilitate understanding of the immunological landscape in pediatric oral RMS, Figure 1 provides a visual summary of the key immunosuppressive components within the TIME, including cellular players and signaling pathways involved in immune evasion.

## 3. Emerging Immunotherapeutic Strategies in Pediatric Rhabdomyosarcoma

Recent insights into the TME and antitumor immune mechanisms have significantly advanced the field of immunotherapy [74]. Dendritic cells (DCs), a subset of antigen-presenting cells (APCs), play a central role in initiating immune responses by engulfing apoptotic cancer cells, processing tumor-associated antigens (TAAs), and presenting them on major histocompatibility complex (MHC) molecules. These antigen-loaded DCs activate T lymphocytes, particularly cytotoxic T cells, which recognize TAAs and eliminate cancer cells. Effective antitumor immunity requires not only antigen presentation but also the delivery of co-stimulatory signals between APCs and effector T cells [27]. Under normal immune surveillance, tumor cells expressing TAAs are typically identified and destroyed. However, during the early stages of tumor development, immune function is often compromised due to mechanisms that suppress immune activation. These include upregulation of immune checkpoints and the recruitment of immunosuppressive cell populations, such as MDSCs and Tregs. Given the critical role of these immune evasion strategies, both the activation of antitumor immunity and the inhibition of immune escape pathways represent promising therapeutic targets across multiple cancer types [27].

Cancer immunotherapy aims to enhance the immune system’s capacity to recognize and eliminate tumor cells, either by directly stimulating immune responses or by increasing tumor immunogenicity to overcome immune evasion mechanisms employed by cancer cells [62]. Several types of immunotherapies have been explored in cancer, such as monoclonal antibodies [75,76], cytokine-based therapies [77], cellular immunotherapies [78], oncolytic viruses [79], and cancer vaccines [80]. However, the effectiveness of these therapies is strongly influenced by the tumor’s immunophenotype, often categorized as either “hot” or “cold” [81]. Cold tumors, which are poorly infiltrated by immune cells, tend to respond better to ex vivo-produced immune components such as recombinant cytokines, tumor-specific monoclonal antibodies, or adoptively transferred immune cells like chimeric antigen receptor (CAR) T cells. In contrast, hot tumors, characterized by a pre-existing, immunologically active TME, are more amenable to strategies that further stimulate immune effector mechanisms. These include vaccination approaches using tumor-associated peptides or autologous DCs, immune checkpoint blockade therapies, and oncolytic viruses that can trigger potent anti-tumor immune responses [82].

As a pediatric malignancy, RMS exhibits a markedly different response to immune-based therapies compared to adult tumors. While the pediatric immune system is generally more adaptable and vigorous than that of adults [83], pediatric cancers, such as RMS, tend to be driven by transcriptional alterations, including chromosomal rearrangements, rather than the accumulation of nonsynonymous genetic mutations [84,85]. Consequently, RMS and other childhood tumors typically have a lower mutational burden and express few or no neoantigens detectable by the immune system [17]. Therefore, the TME in RMS is often more immunosuppressive in pediatric cancers. As a result, many immunotherapies that show efficacy in adult malignancies are frequently ineffective in children [86,87,88].

Immunologically, the RMS microenvironment appears to be relatively “cold.” In a study by Kather JN et al., PD-L1 expressing tumor cells and CD8^+^ PD1^+^ T lymphocytes were identified, although overall CD8^+^ T cell infiltration was notably lower than in other pediatric cancers [89]. Further immunoprofiling revealed that the most abundant myeloid populations in RMS are M2-polarized TAMs and resting M0 macrophages [58]. These M2 TAMs, known for their anti-inflammatory and protumorigenic roles, were also confirmed by a single-cell transcriptomic atlas derived from primary RMS tumors and patient-derived organoid models [36].

Robust preclinical models for RMS have evaluated various modalities, including CAR-T cells, bispecific T cell engagers (BiTEs), and antibody peptide epitope conjugates (APECs) [90,91]. Critical differences in T cell infiltration dynamics, tumor engagement, and cytotoxic activity were identified across these therapies. EGFR-targeted immunotherapies were highlighted as a particularly effective strategy against RMS, offering compelling preclinical support for expanding the evaluation of diverse T cell therapies in this context [91]. Importantly, the application of combination treatments involving standard therapies and targeted immunotherapies holds potential to convert immunologically “cold” RMS tumors into “hot” ones [92]. Strategies that enhance tumor immunogenicity and modulate the immune microenvironment may ultimately improve clinical outcomes for children with RMS.

### 3.1. Antibody-Based Therapies

Monoclonal antibodies (mAbs) exert their antitumor effects by targeting tumor-specific antigens and employing several mechanisms of action. These include blocking oncogenic signaling pathways [93], modulating the host immune response to malignancies [94], delivering cytotoxic agents through antibody-drug conjugates (ADCs) [95], and mediating antibody-dependent cellular cytotoxicity (ADCC) [96]. The effectiveness of mAb-based therapies is highly dependent on identifying antigens that are either uniquely expressed or significantly overexpressed on tumor cells compared to normal tissues, thereby ensuring both efficacy and safety.

### 3.2. Immune Checkpoint Inhibitors

Immune checkpoints such as CTLA-4 and PD-1 act as essential negative regulators of T cell activation and function. Tumor cells, along with other components of the TME, often express ligands for these checkpoints: CD80 for CTLA-4 and PD-L1 for PD-1, which helps tumors evade the immune system by suppressing T cell-mediated antitumor activity [97,98]. Several clinical trials are currently examining the potential of ICIs in soft-tissue sarcomas, including RMS [99].

Studies have evaluated PD-1/PD-L1 expression in pediatric RMS and explored their clinicopathological relevance using immunohistochemistry and tissue microarrays [70,100]. PD-L1 expression was detected on tumor-associated immune cells but not on the RMS tumor cells themselves. Further, PD-L1 levels were found to be elevated in post-chemotherapy samples [100], suggesting that chemotherapy may play a role in rendering RMS tumors more immunogenic or “hot.” A study by Kim et al. evaluated the prognostic significance of PD-L1 expression in several types of soft tissue sarcomas, including 32 RMS patients [101]. PD-L1 positivity was detected in 38% of RMS cases; however, PD-L1 expression was independently associated with poor prognosis [101]. These findings underscore the need for precise immune profiling to identify RMS patients who are more likely to benefit from ICI therapy, particularly in combination with chemotherapy. For instance, in the PEMBROSARC trial in adult soft-tissue sarcoma patients, pembrolizumab (an anti-PD-1 agent) was administered in combination with low-dose cyclophosphamide to patients selected based on the presence of tertiary lymphoid structures (TLS) [102,103]. The trial confirmed TLS as a predictive biomarker for anti-PD-1 therapy in advanced soft-tissue sarcomas [103].

On the other hand, a study on Nivolumab, a PD-1 blocking antibody, in children and young adults with recurrent or refractory solid tumors, including 12 cases of RMS, revealed that there was no significant single-agent activity, consistent with the low PD-L1 expression levels in RMS tumors [104]. Similarly, a phase I trial assessing ipilimumab, an anti-CTLA-4 antibody, in pediatric solid tumors, including RMS cases, showed no objective tumor regressions [105]. However, a case report of a 12-year-old patient with metastatic pleomorphic RMS who, after failing to respond to chemotherapy and antiangiogenic therapy, experienced clinical benefit following treatment with nivolumab [106]. This supports the previous conclusion that, despite the failure of prior treatments, they modulate the TME, thereby enhancing responsiveness to ICI blockade, and further reinforces the rationale for combining ICIs with conventional therapies in RMS.

Integrative proteomics and transcriptomic profiling of RMS samples identified several targetable surface proteins that are highly enriched in RMS. Among these is the immune checkpoint molecule B7-H3 (CD276), which has emerged as a particularly compelling candidate [72,107]. B7-H3, a member of the B7-CD28 family, functions as an immune checkpoint molecule and plays a key role in immune evasion in RMS [72]. Emerging mechanistic evidence highlights B7-H3’s role of B7-H3 in promoting tumor immune evasion and facilitating RMS progression, thereby strengthening the rationale for developing B7-H3-directed immunotherapies for RMS treatment [72]. Preclinical investigations have demonstrated the efficacy of m276-SL-PBD, an antibody-drug conjugate (ADC) targeting B7-H3, in xenograft models of pediatric solid tumors, including RMS [108]. Furthermore, enoblituzumab, a humanized IgG1 monoclonal antibody directed against B7-H3, has been evaluated in children with relapsed or refractory solid tumors exhibiting high B7-H3 expression, including RMS [109].

### 3.3. Cancer Vaccines

Cancer vaccines aim to eliminate minimal residual disease and induce durable, antigen-specific immune memory by stimulating an adaptive T cell response with minimal toxicity. This is achieved through the exogenous administration of tumor-associated antigens (TAAs), which can be delivered in various forms such as DNA, mRNA, peptides, full-length proteins, tumor cell lysates, or autologous DCs, with or without adjuvants [80].

For instance, recombinant human milk peptide lactaptin (RL2) showed an anti-tumor effect in the RMS mouse model by providing long-lasting, immune-mediated protection [110]. Furthermore, inhibition of the immunosuppressive enzyme indoleamine 2,3-dioxygenase (IDO) enhanced the efficacy of RL2-based vaccination, resulting in improved long-term antitumor responses compared to vaccination alone [110]. Additionally, insulin-like growth factor 2 (IGF2), via its receptor IGF-1 receptor 1 (IGFR1), has been identified as suppressing metastatic growth in RMS, establishing IGF2 as a novel immunotherapeutic target [111]. Hence, cells were engineered to co-express human epidermal growth factor receptor 2 (HER2) and IGF1R to develop a cancer cell vaccine [111].

Cancer vaccines can also be delivered using oncolytic viruses, which simultaneously promote tumor cell lysis and activate the immune system. Given the central role of RAS signaling in embryonal RMS (eRMS) pathogenesis [17], a recombinant myxoma virus (MYXV) armed with CRISPR/Cas9 gene-editing tools was engineered to target activated NRAS genes in eRMS tumors specifically. This targeted virotherapy significantly reduced tumor growth and improved overall survival [112]. These findings provide a promising framework for the development of gene-editing-based immunovirotherapies with translational potential in RMS.

Given the pivotal role of DCs in orchestrating antitumor immune responses, several clinical studies have explored DC-based immunotherapy (autologous DCs pulsed with specific tumor-associated antigens, or TAAs) across various malignancies. Several studies have explored the use of standard chemotherapy in combination with DC-based immunotherapy in pediatric sarcomas, including RMS, where only some patients responded to this mode of therapy [113,114]. These findings suggest that only a subset of patients may benefit from DC-based immunotherapy, and that the induction of a robust T-cell response is associated with improved clinical outcomes. Adjuvant strategies aimed at enhancing T-cell activation could potentially augment the efficacy of DC-based approaches. However, most of these studies included only a small number of RMS patients, so larger clinical trials are warranted to validate these findings in the RMS population.

### 3.4. Cellular Immunotherapies

Adoptive cellular immunotherapies involve the manipulation, expansion, and activation of immune cells to elicit a targeted antitumor response. Several immune cell types, including NK cells and CAR T/NK cells, have been employed as therapeutic platforms [115].

#### 3.4.1. CAR-T Cell Therapies

CAR therapies involve the genetic modification of T cells to express synthetic receptors that merge the antigen-recognition capability of antibodies with the cytotoxic functions of T cells. This approach enables T cells to recognize and kill tumor cells in a highly specific, MHC-independent manner. CAR-T cell therapy has revolutionized the treatment of hematologic malignancies, now accounting for over half of all cellular therapies in clinical use or under development [116]. However, they still face substantial challenges in treating solid tumors, including limited T cell infiltration, immunosuppressive TME, and antigen heterogeneity [117,118,119]. Several CAR-T therapies targeting RMS-specific antigens are currently under investigation at both the preclinical and clinical levels.

Another possible approach to targeting B7-H3 in RMS is through directed CAR-T cell therapy, which has led to complete tumor eradication in mouse models, confirming the strong therapeutic potential of this target in RMS [120]. Also, a recent study by Lake et al. reported that IL-8 is upregulated in RMS and osteosarcoma (OS), especially following radiation. Hence, engineering B7-H3 CAR T cells to express the IL-8 receptor CXCR2 significantly improved their tumor infiltration, metabolic activity, and antitumor efficacy. Such CXCR2-enhanced B7-H3 CAR T cells could be a promising therapy for IL-8-producing, B7-H3-expressing solid tumors like RMS and OS [121]. This still needs to be further validated in clinical settings on RMS patients.

One of the earliest RMS-specific CAR-T targets identified was the γ-subunit of the fetal acetylcholine receptor (fAChR), which is minimally expressed in mature muscle after birth but remains in RMS tumors [122]. First-generation fAChR-CAR-T cells showed the secretion of IFN-γ and effective lysis of RMS cells in vitro [123]. However, second-generation CARs with a CD28 costimulatory domain had reduced cytolytic activity, indicating that RMS tumors have some resistance to T cell-mediated killing [124]. This resistance was linked to the absence of essential costimulatory ligands, providing valuable insights into the mechanisms that limit the effectiveness of CAR-T cells [125]. Other promising preclinical results with CAR-T cells have been achieved by targeting IGF1R and receptor tyrosine kinase-like orphan receptor 1 (ROR1), both of which are expressed across many sarcoma cell lines, including RMS. These CAR-T cells significantly slowed the growth of RMS tumors in vitro [126]. Another study focused on CAR-T cells engineered to target the platelet-derived growth factor receptor α (PDGFRA), which is known to play a crucial role in RMS tumor initiation and progression [127]. In their research, PDGFRA CAR-T cells showed strong antitumor activity both in vitro and in subcutaneous xenograft models, producing high levels of immune-stimulatory cytokines and effectively lysing PDGFRA-overexpressing RMS cells [127].

Another promising target is Ephrin type-B receptor 4 (EPHB4), a member of the largest subfamily of receptor tyrosine kinases, which is overexpressed in various malignancies, including RMS [128]. Kubo and colleagues developed a novel CAR construct using EPHRIN B2, the natural ligand of EPHB4, as the targeting domain. These EPHB4-CAR-T cells displayed stable expression and demonstrated potent antitumor efficacy against EPHB4-positive RMS cells both in vitro and in RMS xenograft mouse models, suggesting strong therapeutic potential for RMS treatment [128]. Fibroblast growth factor receptor 4 (FGFR4) has also emerged as an RMS-specific target, which was tested using FGFR4-targeted CAR-T cells. The study reported effective in vitro cytotoxicity and partial tumor control in a disseminated RMS mouse model. However, these CAR-T cells failed to control tumor growth in orthotopic models, which better mimic the human disease [129]. Another study by Alijaj et al. developed FGFR4-targeting CAR-T cells using FGFR4-specific single-domain antibodies (sdAbs). These CAR-T cells exhibited potent and specific cytotoxicity against RMS cells in vitro; however, they were only able to delay tumor growth in orthotopic models [130]. This could be due to the presence of immunosuppressive myeloid cell populations and soluble inhibitory factors in the orthotopic RMS TME that limited FGFR4-CAR-T cell efficacy [120,131]. Hence, a second-generation FGFR4-CAR-T construct was engineered, exhibiting enhanced cytokine production and selective cytotoxicity in vitro. Such a treatment achieved complete elimination of both metastatic and orthotopic RMS tumors in vivo [132]. Additionally, incorporating an inducible caspase-9 (iCasp9) suicide gene into the CAR-T construct of FGFR4 CAR-T cells was investigated to enable the controlled elimination of the cells if necessary. The study findings support the potential of FGFR4 CAR-T cells as a safe and effective immunotherapy for RMS [133]. Another approach involved an optimized FGFR4 CAR-T design utilizing CD28-derived domains, which enhanced antitumor efficacy in most RMS xenograft models. To overcome resistance in aggressive RMS559 cells, the authors developed a bicistronic CAR (BiCisCAR) targeting both FGFR4 and B7-H3, each with distinct co-stimulatory domains. This dual-targeting approach enhanced CAR-T cell persistence and potency, establishing a promising strategy for RMS immunotherapy [45].

Other CAR-T cell therapies for RMS include autologous HER2-targeted CAR-T cells, CD56-targeted CAR-T cell therapy, and C7R-GD2 CAR-T cells [134,135,136]. GD2 is expressed in 55% of pediatric sarcomas, with the highest levels in osteosarcoma and alveolar RMS. CAR.GD2 T-cells demonstrated strong antitumor activity and persistence in preclinical models, particularly when combined with the EZH2 inhibitor Tazemetostat, which upregulated GD2 expression in low-expressing tumors. However, tumor-derived G-CSF promotes the expansion of MDSCs, thereby limiting CAR-T efficacy and highlighting the need for strategies to overcome the immunosuppressive environment [137].

Despite these promising outcomes, CAR-T therapy for solid tumors, including RMS, remains challenging. A significant concern is on-target, off-tumor toxicity, due to shared antigen expression between tumors and healthy tissues. Therefore, advancing CAR-T therapy for RMS requires continued preclinical optimization of CAR design, the identification of truly tumor-specific antigens, and the rational combination of therapies to improve efficacy and safety.

#### 3.4.2. CAR-NK Cells

NK cells are innate immune effectors that can recognize and kill infected or malignant cells without prior sensitization. Their cytotoxic function is mediated through the release of perforin and granzymes, the expression of death receptor ligands, and the secretion of immunostimulatory cytokines [138]. NK cell activity is governed by a dynamic balance between activating and inhibitory receptors, with activation triggered when signals from inhibitory receptors, such as those recognizing MHC-I molecules, are absent or outweighed [115,138]. This is particularly relevant in RMS, where some poorly differentiated tumors exhibit low classical HLA class I expression, rendering them more susceptible to NK cell-mediated cytotoxicity [139,140,141]. Therefore, it is critical to perform tumor immune profiling to identify optimal candidates for NK-based therapies and to guide strategies for NK cell activation, such as cytokine priming. Moreover, the cytotoxic potential of NK cells can be augmented using cytokines [142]. For example, IL-15-treated allogeneic NK cells showed significantly higher cytotoxicity against RMS cell lines compared to resting NK cells [143]. This was further validated in RMS mouse models, where NK cells pretreated with IL-12, IL-15, and/or IL-21 resulted in significant tumor growth inhibition [144,145]. Cytokines such as TNF and IL-1β effectively upregulated immunogenic markers (MHC-I, ICAM-1, CD83, and PD-L1) and enhanced antigen-specific CD8^+^ T cell cytotoxicity against pediatric sarcoma cell lines, including RMS [146]. On the other hand, combining NK cell therapy with antibody-mediated tumor targeting represents another promising strategy. For instance, co-treatment with anti-CXCR4 monoclonal antibodies and activated/expanded NK cells inhibited migration, invasion, and metastasis formation in RMS xenograft models [147]. Further clinical studies using hematopoietic stem cell transplantation (HSCT) or NK cell transfer in RMS patients have been unsuccessful in most studies [148,149]. However, a study by Pérez-Martínez et al. with one RMS patient that showed full donor NK cell chimerism, high cytotoxicity against K562 cells, and achieved complete remission [150].

NK cells can also be genetically modified to express CARs, thereby enhancing their specificity and efficacy. Although CAR-NK cells are more challenging to engineer than CAR-T cells, they offer several advantages: they are better tolerated, can be activated without prior sensitization, and are capable of recognizing and responding to low levels of tumor-associated antigens (TAAs) [151,152]. In RMS, CAR-NK cells targeting HER2 have demonstrated potent antitumor activity in vitro. Furthermore, a study by Gossel et al. utilized NK-92 cell lines to develop HER2-specific CAR-engineered NK-92 cells, which exhibited high and selective cytotoxicity against RMS cells [153]. A study by Lam et al. developed EphA2-targeted CAR-NK cells using mRNA electroporation, achieving stable CAR expression and enhanced cytotoxicity against RMS cell lines. In vivo, CAR-NK cells demonstrated significant anti-tumor activity in RMS [154]. Furthermore, chemotherapeutic agent bortezomib was reported to sensitize relapsed/refractory RMS cells and tumor organoids to apoptosis by upregulating the TRAIL receptor DR5. When combined with NK-92/5.28.z CAR-NK cells or recombinant TRAIL, the treatment significantly enhanced antitumor activity via activation of NF-κB, JNK, and caspase pathways. These findings support the use of bortezomib to potentiate TRAIL- and CAR-mediated cytotoxicity in resistant RMS tumors [155].

#### 3.4.3. CAR-Cytokine Induced Killer (CIK) Cells

Cytokine-induced killer (CIK) cells represent a heterogeneous population of polyclonal T lymphocytes that acquire phenotypic characteristics and cytotoxic functions resembling those of NK cells through specific culture conditions [156]. While most CAR-engineering strategies have focused on conventional T cells, modifying CIK cells with CAR constructs has also yielded promising preclinical outcomes [157,158], particularly against HER2/ERBB2-expressing RMS [158]. Another study developed second-generation ERBB2-targeted CAR-CIK cells to treat disseminated high-risk alveolar RMS in a xenograft model [159]. These CAR-CIK cells demonstrated superior specificity and cytotoxicity against ERBB2-positive tumor cells compared to wild-type CIK cells. Notably, CAR-CIK therapy led to the accumulation of NK and NKT cell subpopulations in distant tumor sites, enhancing innate immune responses [159]. These findings position CAR-CIK cells as a powerful immunotherapeutic strategy for RMS, combining targeted cytotoxicity with innate immune modulation. CAR-CIK cells exhibited potent cytotoxicity against RMS cell lines and primary tumors, with enhanced expansion, cytokine secretion (e.g., IFN-γ, perforin), and activation of cytotoxic pathways. In vivo, a single dose of CAR-CIK cells significantly reduced metastasis and improved survival in an RMS xenograft model. These results indicate that CAR-CIK cells are at least as effective as CAR-T cells and offer additional advantages in terms of safety and potential for allogeneic use, supporting their promise for treating solid tumors [160].

Key Remark: While novel immunotherapies, such as CAR-T, CAR-NK, and checkpoint inhibitors, hold promise, their efficacy in pediatric oral RMS remains limited by poor immune infiltration and immunosuppressive feedback loops. Combination strategies and improved immune engineering may enhance therapeutic outcomes.

**Figure 2:**
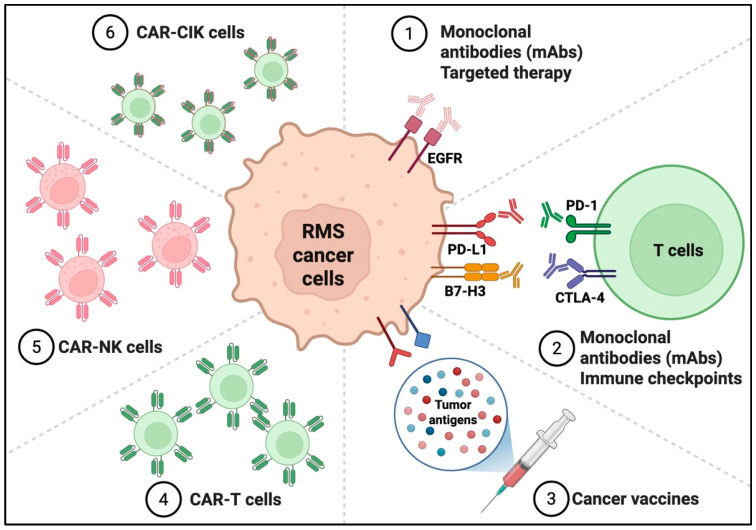
Emerging Immunotherapeutic Strategies in Pediatric Oral Rhabdomyosarcoma.

Figure 2 outlines the current and emerging immunotherapeutic approaches being investigated for pediatric oral RMS, with a focus on cellular therapies, immune checkpoint blockade, and innovative combinatorial strategies.

## 4. Challenges and Future Directions

Although promising advances have been made in immunotherapeutic strategies for pediatric oral RMS, several key challenges still hinder their clinical success and broader adoption. These obstacles are multifaceted, involving biological complexities specific to pediatric tumors, technical issues with therapeutic delivery, ethical concerns in treating children, and the urgent need for reliable biomarkers to support precision medicine approaches [161,162].

### 4.1. Integrating Immunotherapy with Standard Treatments

The integration of immunotherapies, such as checkpoint inhibitors or CAR-T cell therapy, with existing chemotherapy and radiation therapy regimens presents both opportunities and challenges. Standard treatments often cause immunosuppressive effects, including loss of lymphocytes and cytokine dysregulation, which can potentially diminish the effectiveness of immunotherapies [29]. Conversely, some chemoradiation strategies may enhance tumor antigen presentation and induce immune-activating cell death, thus creating a combined approach for immuno-oncology. However, the optimal timing, dosing, and combination strategies are still under active investigation, especially in pediatric patients with developing immune systems [163].

### 4.2. Biological and Developmental Barriers in Pediatric Tumors

Unlike adult cancers, pediatric RMS frequently shows a low TMB, limited neoantigen expression, and a generally “immune-cold” TME. These elements result in poor immunogenicity and a less effective response to checkpoint blockade therapies. Additionally, fusion-positive RMS subtypes, such as those with PAX3:FOXO1 rearrangements, actively inhibit antigen processing and MHC-I expression, further aiding immune evasion. Since pediatric tumors exhibit developmental differences, further research is needed to understand how immune checkpoint regulation occurs during development and how to target the tumor’s immunoediting abilities without harming normal tissue development [164,165].

### 4.3. Technical and Ethical Constraints in Pediatric Immunotherapy

Technical limitations, such as cell manufacturing scalability, T cell exhaustion, and cytokine release syndrome (CRS), become more pronounced in pediatric settings where patient weight, immune maturity, and tolerance to systemic toxicity vary greatly [166]. Ethical concerns also impose significant restrictions. The balance between therapeutic innovation and the ethical imperative to “not harm” is more delicate in pediatric populations, often causing regulatory and logistical delays in trial implementation [167,168].

### 4.4. The Biomarker Gap: Stratifying Patients for Response

A significant unmet need is the identification of predictive biomarkers to classify patients who are likely to benefit from immunotherapy. For example, expression levels of PD-L1, CD276 (B7-H3), or tumor-infiltrating lymphocyte (TIL) profiles could serve as early indicators of treatment success. However, these biomarkers have not been validated in large pediatric RMS groups, and inter-tumoral heterogeneity remains a confounding factor [109]. Single-cell RNA sequencing and spatial transcriptomics could enable more precise patient stratification by uncovering immune niches and cellular interactions within the TME [36].

### 4.5. Personalizing Immuno-Oncology for Pediatric Rhabdomyosarcoma

The future of RMS immunotherapy relies on precision and personalization. Combining immunotherapy with epigenetic modulators, targeted therapies (e.g., FGFR4 and CXCR4 inhibitors), and agents that reprogram metabolism could significantly transform the immune landscape. Additionally, personalized cancer vaccines based on neoantigen profiling and adoptive T cell therapies targeting patient-specific tumor antigens may redefine treatment standards [169]. Incorporating these strategies with real-time immune monitoring and adaptive clinical trial designs will be crucial [170].

### 4.6. Looking Forward: Translational and Collaborative Imperatives

To translate immuno-oncology research into clinical practice for pediatric RMS, interdisciplinary collaboration is essential. Ongoing efforts, such as the INFORM registry and Pediatric MATCH trial, are beginning to connect molecular profiling with immunotherapy eligibility [171,172]. Future studies must ensure fair inclusion of all pediatric RMS subtypes, employ comprehensive immunophenotyping, and emphasize long-term follow-up to track late toxicities. Additionally, international consortia should be used to pool resources and develop statistically robust datasets that can inform evidence-based treatment decisions [173].

Key Remark: Progress in pediatric RMS immunotherapy requires age-specific approaches, ethical sensitivity, and precision medicine tools. Emphasis should be placed on immune profiling, rational combinations with standard therapy, and development of robust pediatric immuno-oncology clinical trial frameworks.

## 5. Conclusions

Pediatric RMS remains a rare but aggressive malignancy marked by a profoundly immunosuppressive TIME dominated by M2-polarized macrophages, regulatory T cells, and myeloid-derived suppressor cells. These immune cells, combined with mechanisms such as T cell exhaustion, a low neoantigen burden, and downregulation of MHC class I expression, collectively impair anti-tumor immunity, positioning pediatric RMS as an “immune-cold” tumor with limited responsiveness to conventional immunotherapies. Emerging immunotherapeutic strategies, including CAR-T, CAR-NK, and CAR-CIK cell therapies; checkpoint inhibitors; oncolytic viruses; and cancer vaccines, show promise in overcoming these barriers. However, their clinical translation remains constrained by challenges such as poor immune cell infiltration, antigen heterogeneity, developmental and ethical considerations in pediatric patients, and the absence of validated predictive biomarkers. Notably, combinatorial approaches that integrate immunotherapy with conventional treatments, epigenetic modulators, or metabolic reprogramming agents may enhance antigen presentation, reprogram the TIME, and improve immune cell trafficking into tumors. Future research should prioritize biomarker-driven patient selection, age-specific immune profiling, and adaptive clinical trial designs that accommodate the biological and ethical complexities of pediatric oncology. Rational combination therapies that aimed at converting “cold” tumors into “hot” immune phenotypes hold particular promise for improving response rates and long-term outcomes. A multidisciplinary, collaborative approach, incorporating immunology, oncology, pediatrics, and bioengineering, will be essential for translating laboratory findings into safe, effective, and durable immunotherapeutic strategies that improve survival while preserving quality of life in children with RMS.

## Figures and Tables

**Table 1:** Summarizes the current therapeutic modalities for pediatric Oral RMS [1,2,3,4,8,9,10,13,14,15].

Modality	Role in RMS Treatment	Limitations
Surgery	Local tumor control by complete excision.	Often limited by anatomical complexity in the oral cavity; may affect function and appearance.
Chemotherapy	Systemic disease control; commonly includes vincristine, actinomycin D, and cyclophosphamide (VAC regimen).	Systemic toxicity, resistance in high-risk subtypes.
Radiotherapy	Local control for unresectable or residual disease.	Growth inhibition, craniofacial deformities, cognitive effects in children.
Targeted Therapy	Includes inhibitors of IGF1R, FGFR4, and other molecular targets relevant to RMS biology.	Target specificity and resistance; mostly in experimental stages.
Immunotherapy	Emerging approaches include CAR-T, checkpoint inhibitors, and oncolytic viruses; efficacy in pediatric RMS under investigation.	Limited clinical data in children; immune ‘coldness’ of tumors poses challenge.

## References

[1] Pathania A.S. (2024). Immune Microenvironment in Childhood Cancers: Characteristics and Therapeutic Challenges. Cancers.

[2] Bosse K.R., Majzner R.G., Mackall C.L., Maris J.M. (2020). Immune-Based Approaches for the Treatment of Pediatric Malignancies. Annu. Rev. Cancer Biol..

[3] Hutzen B., Paudel S.N., Naeimi Kararoudi M., Cassady K.A., Lee D.A., Cripe T.P. (2019). Immunotherapies for pediatric cancer: Current landscape and future perspectives. Cancer Metastasis Rev..

[4] Malempati S., Hawkins D.S. (2012). Rhabdomyosarcoma: Review of the Children’s Oncology Group (COG) Soft-Tissue Sarcoma Committee experience and rationale for current COG studies. Pediatr. Blood Cancer.

[5] Agaram N.P. (2022). Evolving classification of rhabdomyosarcoma. Histopathology.

[6] Ognjanovic S., Linabery A.M., Charbonneau B., Ross J.A. (2009). Trends in childhood rhabdomyosarcoma incidence and survival in the United States, 1975–2005. Cancer.

[7] Rudzinski E.R., Anderson J.R., Chi Y.Y., Gastier-Foster J.M., Astbury C., Barr F.G., Skapek S.X., Hawkins D.S., Weigel B.J., Pappo A. (2017). Histology, fusion status, and outcome in metastatic rhabdomyosarcoma: A report from the Children’s Oncology Group. Pediatr. Blood Cancer.

[8] Chen C., Garcia H.D., Scheer M., Henssen A.G. (2019). Current and Future Treatment Strategies for Rhabdomyosarcoma. Front. Oncol..

[9] Van Tilburg C.M., Pfaff E., Pajtler K.W., Langenberg K.P.S., Fiesel P., Jones B.C., Balasubramanian G.P., Stark S., Johann P.D., Blattner-Johnson M. (2021). The Pediatric Precision Oncology INFORM Registry: Clinical Outcome and Benefit for Patients with Very High-Evidence Targets. Cancer Discov..

[10] Dasgupta R., Fuchs J., Rodeberg D. (2016). Rhabdomyosarcoma. Semin. Pediatr. Surg..

[11] Crist W.M., Anderson J.R., Meza J.L., Fryer C., Raney R.B., Ruymann F.B., Breneman J., Qualman S.J., Wiener E., Wharam M. (2001). Intergroup rhabdomyosarcoma study-IV: Results for patients with nonmetastatic disease. J. Clin. Oncol..

[12] Dinakar J., Gowri S., Tryphena E.T.A. (2023). Alveolar type of rhabdomyosarcoma of maxilla-A case report. J. Oral Maxillofac. Pathol..

[13] Skapek S.X., Ferrari A., Gupta A.A., Lupo P.J., Butler E., Shipley J., Barr F.G., Hawkins D.S. (2019). Rhabdomyosarcoma. Nat. Rev. Dis. Primers.

[14] Rogers T.N., Dasgupta R. (2021). Management of Rhabdomyosarcoma in Pediatric Patients. Surg. Oncol. Clin. N. Am..

[15] Raney R.B., Maurer H.M., Anderson J.R., Andrassy R.J., Donaldson S.S., Qualman S.J., Wharam M.D., Wiener E.S., Crist W.M. (2001). The Intergroup Rhabdomyosarcoma Study Group (IRSG): Major Lessons From the IRS-I Through IRS-IV Studies as Background for the Current IRS-V Treatment Protocols. Sarcoma.

[16] Crompton B.D., Stewart C., Taylor-Weiner A., Alexe G., Kurek K.C., Calicchio M.L., Kiezun A., Carter S.L., Shukla S.A., Mehta S.S. (2014). The genomic landscape of pediatric Ewing sarcoma. Cancer Discov..

[17] Shern J.F., Chen L., Chmielecki J., Wei J.S., Patidar R., Rosenberg M., Ambrogio L., Auclair D., Wang J., Song Y.K. (2014). Comprehensive genomic analysis of rhabdomyosarcoma reveals a landscape of alterations affecting a common genetic axis in fusion-positive and fusion-negative tumors. Cancer Discov..

[18] Chen D.S., Mellman I. (2017). Elements of cancer immunity and the cancer-immune set point. Nature.

[19] Wessel K.M., Kaplan R.N. (2022). Targeting tumor microenvironment and metastasis in children with solid tumors. Curr. Opin. Pediatr..

[20] Singh S., Abu-Zaid A., Jin H., Fang J., Wu Q., Wang T., Feng H., Quarni W., Shao Y., Maxham L. (2022). Targeting KDM4 for treating PAX3-FOXO1-driven alveolar rhabdomyosarcoma. Sci. Transl. Med..

[21] Thorsson V., Gibbs D.L., Brown S.D., Wolf D., Bortone D.S., Ou Yang T.H., Porta-Pardo E., Gao G.F., Plaisier C.L., Eddy J.A. (2018). The Immune Landscape of Cancer. Immunity.

[22] Emens L.A., Romero P.J., Anderson A.C., Bruno T.C., Capitini C.M., Collyar D., Gulley J.L., Hwu P., Posey A.D., Silk A.W. (2024). Challenges and opportunities in cancer immunotherapy: A Society for Immunotherapy of Cancer (SITC) strategic vision. J. Immunother. Cancer.

[23] Sherif S., Roelands J., Mifsud W., Ahmed E.I., Raynaud C.M., Rinchai D., Sathappan A., Maaz A., Saleh A., Ozer E. (2022). The immune landscape of solid pediatric tumors. J. Exp. Clin. Cancer Res..

[24] Terry R.L., Meyran D., Fleuren E.D.G., Mayoh C., Zhu J., Omer N., Ziegler D.S., Haber M., Darcy P.K., Trapani J.A. (2021). Chimeric Antigen Receptor T cell Therapy and the Immunosuppressive Tumor Microenvironment in Pediatric Sarcoma. Cancers.

[25] Gomez S., Tabernacki T., Kobyra J., Roberts P., Chiappinelli K.B. (2020). Combining epigenetic and immune therapy to overcome cancer resistance. Semin. Cancer Biol..

[26] De la Nava D., Selvi K.M., Alonso M.M. (2022). Immunovirotherapy for Pediatric Solid Tumors: A Promising Treatment That is Becoming a Reality. Front. Immunol..

[27] Miwa S., Yamamoto N., Hayashi K., Takeuchi A., Igarashi K., Tsuchiya H. (2020). Recent Advances and Challenges in the Treatment of Rhabdomyosarcoma. Cancers.

[28] Olsen H.E., Lynn G.M., Valdes P.A., Cerecedo Lopez C.D., Ishizuka A.S., Arnaout O., Bi W.L., Peruzzi P.P., Chiocca E.A., Friedman G.K. (2021). Therapeutic cancer vaccines for pediatric malignancies: Advances, challenges, and emerging technologies. Neuro-Oncol. Adv..

[29] Morel V.J., Rossler J., Bernasconi M. (2024). Targeted immunotherapy and nanomedicine for rhabdomyosarcoma: The way of the future. Med. Res. Rev..

[30] Panagi M., Pilavaki P., Constantinidou A., Stylianopoulos T. (2022). Immunotherapy in soft tissue and bone sarcoma: Unraveling the barriers to effectiveness. Theranostics.

[31] Arneth B. (2019). Tumor Microenvironment. Medicina.

[32] Anderson N.M., Simon M.C. (2020). The tumor microenvironment. Curr. Biol..

[33] Chaudhary B., Elkord E. (2016). Regulatory T Cells in the Tumor Microenvironment and Cancer Progression: Role and Therapeutic Targeting. Vaccines.

[34] Strizova Z., Benesova I., Bartolini R., Novysedlak R., Cecrdlova E., Foley L.K., Striz I. (2023). M1/M2 macrophages and their overlaps—Myth or reality?. Clin. Sci..

[35] Peng Y., Zhou M., Yang H., Qu R., Qiu Y., Hao J., Bi H., Guo D. (2023). Regulatory Mechanism of M1/M2 Macrophage Polarization in the Development of Autoimmune Diseases. Mediat. Inflamm..

[36] DeMartino J., Meister M.T., Visser L.L., Brok M., Groot Koerkamp M.J.A., Wezenaar A.K.L., Hiemcke-Jiwa L.S., de Souza T., Merks J.H.M., Rios A.C. (2023). Single-cell transcriptomics reveals immune suppression and cell states predictive of patient outcomes in rhabdomyosarcoma. Nat. Commun..

[37] Rutland C.D., Gedallovich J., Wang A., Zdravkovic S., Varma S., Hornick J.L., Charville G.W. (2023). Diagnostic utility of FOXO1 immunohistochemistry for rhabdomyosarcoma classification. Histopathology.

[38] Feng G., Guo Y., Chen M., Zhang Y., Liu Z., Sun C., Hu X., Lin C., Liu Y., Wu Y. (2025). Schwann Cell-Mediated M2-Like Macrophage Polarization in Rhabdomyosarcoma. Oral Dis..

[39] Munisamy S., Radhakrishnan A.K., Ramdas P., Samuel P.J., Singh V.A. (2022). Immune Biomarkers in Blood from Sarcoma Patients: A Pilot Study. Curr. Oncol..

[40] Gutkin D.W., Shurin M.R. (2014). Clinical evaluation of systemic and local immune responses in cancer: Time for integration. Cancer Immunol. Immunother..

[41] Zhang Y., Guo J., Jia R. (2021). Treg: A Promising Immunotherapeutic Target in Oral Diseases. Front. Immunol..

[42] Surendran S., Aboelkheir U., Tu A.A., Magner W.J., Sigurdson S.L., Merzianu M., Hicks W.L., Suresh A., Kirkwood K.L., Kuriakose M.A. (2022). T-Cell Infiltration and Immune Checkpoint Expression Increase in Oral Cavity Premalignant and Malignant Disorders. Biomedicines.

[43] Maggi E., Munari E., Landolina N., Mariotti F.R., Azzarone B., Moretta L. (2024). T cell landscape in the microenvironment of human solid tumors. Immunol. Lett..

[44] Ozaniak A., Vachtenheim J., Lischke R., Bartunkova J., Strizova Z. (2021). Novel Insights into the Immunotherapy of Soft Tissue Sarcomas: Do We Need a Change of Perspective?. Biomedicines.

[45] Tian M., Wei J.S., Cheuk A.T., Milewski D., Zhang Z., Kim Y.Y., Chou H.C., Liu C., Badr S., Pope E.G. (2024). CAR T-cells targeting FGFR4 and CD276 simultaneously show potent antitumor effect against childhood rhabdomyosarcoma. Nat. Commun..

[46] Davicioni E., Anderson J.R., Buckley J.D., Meyer W.H., Triche T.J. (2010). Gene expression profiling for survival prediction in pediatric rhabdomyosarcomas: A report from the children’s oncology group. J. Clin. Oncol..

[47] Kather J.N., Horner C., Weis C.A., Aung T., Vokuhl C., Weiss C., Scheer M., Marx A., Simon-Keller K. (2019). CD163^+^ immune cell infiltrates and presence of CD54^+^ microvessels are prognostic markers for patients with embryonal rhabdomyosarcoma. Sci. Rep..

[48] Lee K.Y., Wong H.Y., Zeng Q., Le Lin J., Cheng M.S., Kuick C.H., Chang K.T.E., Loh A.H.P., Schwarz H. (2021). Ectopic CD137 expression by rhabdomyosarcoma provides selection advantages but allows immunotherapeutic targeting. Oncoimmunology.

[49] Preglej T., Ellmeier W. (2022). CD4(+) Cytotoxic T cells—Phenotype, Function and Transcriptional Networks Controlling Their Differentiation Pathways. Immunol. Lett..

[50] Xie N., Shen G., Gao W., Huang Z., Huang C., Fu L. (2023). Neoantigens: Promising targets for cancer therapy. Signal Transduct. Target. Ther..

[51] Belgiovine C., Mebelli K., Raffaele A., De Cicco M., Rotella J., Pedrazzoli P., Zecca M., Riccipetitoni G., Comoli P. (2024). Pediatric Solid Cancers: Dissecting the Tumor Microenvironment to Improve the Results of Clinical Immunotherapy. Int. J. Mol. Sci..

[52] Chen H., Sameshima J., Yokomizo S., Sueyoshi T., Nagano H., Miyahara Y., Sakamoto T., Fujii S., Kiyoshima T., Guy T. (2023). Expansion of CD4^+^ cytotoxic T lymphocytes with specific gene expression patterns may contribute to suppression of tumor immunity in oral squamous cell carcinoma: Single-cell analysis and in vitro experiments. Front. Immunol..

[53] Li K., Shi H., Zhang B., Ou X., Ma Q., Chen Y., Shu P., Li D., Wang Y. (2021). Myeloid-derived suppressor cells as immunosuppressive regulators and therapeutic targets in cancer. Signal Transduct. Target. Ther..

[54] He S., Zheng L., Qi C. (2025). Myeloid-derived suppressor cells (MDSCs) in the tumor microenvironment and their targeting in cancer therapy. Mol. Cancer.

[55] Youn J.I., Nagaraj S., Collazo M., Gabrilovich D.I. (2008). Subsets of myeloid-derived suppressor cells in tumor-bearing mice. J. Immunol..

[56] Highfill S.L., Cui Y., Giles A.J., Smith J.P., Zhang H., Morse E., Kaplan R.N., Mackall C.L. (2014). Disruption of CXCR2-mediated MDSC tumor trafficking enhances anti-PD1 efficacy. Sci. Transl. Med..

[57] Nomikos J., Wunker C., Waspe A.C., Babichev Y., Piorkowska K., Wong S., Foltz W., Gerstle J.T., Demicco E.G., Gupta A.A. (2024). Abstract A064 Characterizing the immune microenvironment and examining the effect of tumour-targeted MRgHIFU mediated hyperthermia in combination with thermosensitive liposomal doxorubicin in a mouse model of embryonal rhabdomyosarcoma. Cancer Res..

[58] Chen L., Oke T., Siegel N., Cojocaru G., Tam A.J., Blosser R.L., Swailes J., Ligon J.A., Lebid A., Morris C. (2020). The Immunosuppressive Niche of Soft-Tissue Sarcomas is Sustained by Tumor-Associated Macrophages and Characterized by Intratumoral Tertiary Lymphoid Structures. Clin. Cancer Res..

[59] Bien E., Krawczyk M., Izycka-Swieszewska E., Trzonkowski P., Kazanowska B., Adamkiewicz-Drozynska E., Balcerska A. (2013). Deregulated systemic IL-10/IL-12 balance in advanced and poor prognosis paediatric soft tissue sarcomas. Biomarkers.

[60] Sato T., Terai M., Tamura Y., Alexeev V., Mastrangelo M.J., Selvan S.R. (2011). Interleukin 10 in the tumor microenvironment: A target for anticancer immunotherapy. Immunol. Res..

[61] Haque S., Morris J.C. (2017). Transforming growth factor-beta: A therapeutic target for cancer. Hum. Vaccines Immunother..

[62] Mellman I., Coukos G., Dranoff G. (2011). Cancer immunotherapy comes of age. Nature.

[63] Zhang Z., Zhou X., Guo J., Zhang F., Qian Y., Wang G., Duan M., Wang Y., Zhao H., Yang Z. (2022). TA-MSCs, TA-MSCs-EVs, MIF: Their crosstalk in immunosuppressive tumor microenvironment. J. Transl. Med..

[64] Zhang Y., Katkhada K., Meng L.Z., Zhao B., Tong S., Chaabane W., Kallai A., Tobin N.P., Ostman A., Mega A. (2025). Myogenic IGFBP5 levels in rhabdomyosarcoma are nourished by mesenchymal stromal cells and regulate growth arrest and apoptosis. Cell Commun. Signal..

[65] Drouillard D., Craig B.T., Dwinell M.B. (2023). Physiology of chemokines in the cancer microenvironment. Am. J. Physiol. Cell Physiol..

[66] Wu Y., Yi M., Niu M., Mei Q., Wu K. (2022). Myeloid-derived suppressor cells: An emerging target for anticancer immunotherapy. Mol. Cancer.

[67] Dahmani A., Delisle J.S. (2018). TGF-β in T Cell Biology: Implications for Cancer Immunotherapy. Cancers.

[68] Quamine A.E., Olsen M.R., Cho M.M., Capitini C.M. (2021). Approaches to Enhance Natural Killer Cell-Based Immunotherapy for Pediatric Solid Tumors. Cancers.

[69] Milewski D., Tian M., Kim Y., Wei J., Khan J. (2023). Abstract 6736: Suppression of antigen presentation is a hallmark of pediatric rhabdomyosarcoma. Cancer Res..

[70] Gabrych A., Peksa R., Kunc M., Krawczyk M., Izycka-Swieszewska E., Biernat W., Bien E. (2019). The PD-L1/PD-1 axis expression on tumor-infiltrating immune cells and tumor cells in pediatric rhabdomyosarcoma. Pathol. Res. Pract..

[71] Zhang H., Pang Y., Yi L., Wang X., Wei P., Wang H., Lin S. (2025). Epigenetic regulators combined with tumour immunotherapy: Current status and perspectives. Clin. Epigenetics.

[72] Lavoie R.R., Gargollo P.C., Ahmed M.E., Kim Y., Baer E., Phelps D.A., Charlesworth C.M., Madden B.J., Wang L., Houghton P.J. (2021). Surfaceome Profiling of Rhabdomyosarcoma Reveals B7-H3 as a Mediator of Immune Evasion. Cancers.

[73] Getu A.A., Tigabu A., Zhou M., Lu J., Fodstad O., Tan M. (2023). New frontiers in immune checkpoint B7-H3 (CD276) research and drug development. Mol. Cancer.

[74] Miwa S., Nishida H., Tsuchiya H. (2017). Current status of immunotherapy for sarcomas. Immunotherapy.

[75] Weiner L.M., Surana R., Wang S. (2010). Monoclonal antibodies: Versatile platforms for cancer immunotherapy. Nat. Rev. Immunol..

[76] Tsuchikama K., Anami Y., Ha S.Y.Y., Yamazaki C.M. (2024). Exploring the next generation of antibody–drug conjugates. Nat. Rev. Clin. Oncol..

[77] Propper D.J., Balkwill F.R. (2022). Harnessing cytokines and chemokines for cancer therapy. Nat. Rev. Clin. Oncol..

[78] Rosenberg S.A., Restifo N.P., Yang J.C., Morgan R.A., Dudley M.E. (2008). Adoptive cell transfer: A clinical path to effective cancer immunotherapy. Nat. Rev. Cancer.

[79] Kaufman H.L., Kohlhapp F.J., Zloza A. (2015). Oncolytic viruses: A new class of immunotherapy drugs. Nat. Rev. Drug Discov..

[80] Saxena M., van der Burg S.H., Melief C.J.M., Bhardwaj N. (2021). Therapeutic cancer vaccines. Nat. Rev. Cancer.

[81] Wang L., Geng H., Liu Y., Liu L., Chen Y., Wu F., Liu Z., Ling S., Wang Y., Zhou L. (2023). Hot and cold tumors: Immunological features and the therapeutic strategies. MedComm.

[82] Ren X., Guo S., Guan X., Kang Y., Liu J., Yang X. (2022). Immunological Classification of Tumor Types and Advances in Precision Combination Immunotherapy. Front. Immunol..

[83] Simon A.K., Hollander G.A., McMichael A. (2015). Evolution of the immune system in humans from infancy to old age. Proc. Biol. Sci..

[84] Gröbner S.N., Worst B.C., Weischenfeldt J., Buchhalter I., Kleinheinz K., Rudneva V.A., Johann P.D., Balasubramanian G.P., Segura-Wang M., Brabetz S. (2018). The landscape of genomic alterations across childhood cancers. Nature.

[85] Brien G.L., Stegmaier K., Armstrong S.A. (2019). Targeting chromatin complexes in fusion protein-driven malignancies. Nat. Rev. Cancer.

[86] Dyson K.A., Stover B.D., Grippin A., Mendez-Gomez H.R., Lagmay J., Mitchell D.A., Sayour E.J. (2019). Emerging trends in immunotherapy for pediatric sarcomas. J. Hematol. Oncol..

[87] Casey D.L., Cheung N.V. (2020). Immunotherapy of Pediatric Solid Tumors: Treatments at a Crossroads, with an Emphasis on Antibodies. Cancer Immunol. Res..

[88] Long A.H., Morgenstern D.A., Leruste A., Bourdeaut F., Davis K.L. (2022). Checkpoint Immunotherapy in Pediatrics: Here, Gone, and Back Again. American Society of Clinical Oncology Educational Book.

[89] Chowdhury F., Dunn S., Mitchell S., Mellows T., Ashton-Key M., Gray J.C. (2015). PD-L1 and CD8^+^PD1^+^ lymphocytes exist as targets in the pediatric tumor microenvironment for immunomodulatory therapy. OncoImmunology.

[90] Wang Y., Shelton S.E., Kastrunes G., Barbie D.A., Freeman G.J., Marasco W.A. (2022). Preclinical models for development of immune-oncology therapies. Immuno-Oncol. Insights.

[91] Yan C., Yang Q., Zhang S., Millar D.G., Alpert E.J., Do D., Veloso A., Brunson D.C., Drapkin B.J., Stanzione M. (2021). Single-cell imaging of T cell immunotherapy responses in vivo. J. Exp. Med..

[92] Rytlewski J., Milhem M.M., Monga V. (2021). Turning ‘Cold’ tumors ‘Hot’: Immunotherapies in sarcoma. Ann. Transl. Med..

[93] Jacob W., James I., Hasmann M., Weisser M. (2018). Clinical development of HER3-targeting monoclonal antibodies: Perils and progress. Cancer Treat. Rev..

[94] Ferrara N., Hillan K.J., Gerber H.P., Novotny W. (2004). Discovery and development of bevacizumab, an anti-VEGF antibody for treating cancer. Nat. Rev. Drug Discov..

[95] Verma S., Miles D., Gianni L., Krop I.E., Welslau M., Baselga J., Pegram M., Oh D.Y., Diéras V., Guardino E. (2012). Trastuzumab emtansine for HER2-positive advanced breast cancer. N. Engl. J. Med..

[96] Hubert P., Amigorena S. (2012). Antibody-dependent cell cytotoxicity in monoclonal antibody-mediated tumor immunotherapy. Oncoimmunology.

[97] Wei S.C., Levine J.H., Cogdill A.P., Zhao Y., Anang N.A.S., Andrews M.C., Sharma P., Wang J., Wargo J.A., Pe’er D. (2017). Distinct Cellular Mechanisms Underlie Anti-CTLA-4 and Anti-PD-1 Checkpoint Blockade. Cell.

[98] Pardoll D.M. (2012). The blockade of immune checkpoints in cancer immunotherapy. Nat. Rev. Cancer.

[99] Saerens M., Brusselaers N., Rottey S., Decruyenaere A., Creytens D., Lapeire L. (2021). Immune checkpoint inhibitors in treatment of soft-tissue sarcoma: A systematic review and meta-analysis. Eur. J. Cancer.

[100] Bertolini G., Bergamaschi L., Ferrari A., Renne S.L., Collini P., Gardelli C., Barisella M., Centonze G., Chiaravalli S., Paolino C. (2018). PD-L1 assessment in pediatric rhabdomyosarcoma: A pilot study. BMC Cancer.

[101] Kim C., Kim E.K., Jung H., Chon H.J., Han J.W., Shin K.H., Hu H., Kim K.S., Choi Y.D., Kim S. (2016). Prognostic implications of PD-L1 expression in patients with soft tissue sarcoma. BMC Cancer.

[102] Petitprez F., de Reyniès A., Keung E.Z., Chen T.W., Sun C.M., Calderaro J., Jeng Y.M., Hsiao L.P., Lacroix L., Bougoüin A. (2020). B cells are associated with survival and immunotherapy response in sarcoma. Nature.

[103] Italiano A., Bessede A., Pulido M., Bompas E., Piperno-Neumann S., Chevreau C., Penel N., Bertucci F., Toulmonde M., Bellera C. (2022). Pembrolizumab in soft-tissue sarcomas with tertiary lymphoid structures: A phase 2 PEMBROSARC trial cohort. Nat. Med..

[104] Davis K.L., Fox E., Merchant M.S., Reid J.M., Kudgus R.A., Liu X., Minard C.G., Voss S., Berg S.L., Weigel B.J. (2020). Nivolumab in children and young adults with relapsed or refractory solid tumours or lymphoma (ADVL1412): A multicentre, open-label, single-arm, phase 1-2 trial. Lancet Oncol..

[105] Merchant M.S., Wright M., Baird K., Wexler L.H., Rodriguez-Galindo C., Bernstein D., Delbrook C., Lodish M., Bishop R., Wolchok J.D. (2016). Phase I Clinical Trial of Ipilimumab in Pediatric Patients with Advanced Solid Tumors. Clin. Cancer Res..

[106] Liu J., Liu P., Gong F., Tian Y., Zhao X. (2022). Case Report: A PD-L1-Positive Patient With Pleomorphic Rhabdomyosarcoma Achieving an Impressive Response to Immunotherapy. Front. Immunol..

[107] Timpanaro A., Piccand C., Uldry A.C., Bode P.K., Dzhumashev D., Sala R., Heller M., Rössler J., Bernasconi M. (2023). Surfaceome Profiling of Cell Lines and Patient-Derived Xenografts Confirm FGFR4, NCAM1, CD276, and Highlight AGRL2, JAM3, and L1CAM as Surface Targets for Rhabdomyosarcoma. Int. J. Mol. Sci..

[108] Kendsersky N.M., Lindsay J., Kolb E.A., Smith M.A., Teicher B.A., Erickson S.W., Earley E.J., Mosse Y.P., Martinez D., Pogoriler J. (2021). The B7-H3-Targeting Antibody-Drug Conjugate m276-SL-PBD Is Potently Effective Against Pediatric Cancer Preclinical Solid Tumor Models. Clin. Cancer Res..

[109] Rasic P., Jeremic M., Jeremic R., Dusanovic Pjevic M., Rasic M., Djuricic S.M., Milickovic M., Vukadin M., Mijovic T., Savic D. (2023). Targeting B7-H3—A Novel Strategy for the Design of Anticancer Agents for Extracranial Pediatric Solid Tumors Treatment. Molecules.

[110] Troitskaya O., Varlamov M., Nushtaeva A., Richter V., Koval O. (2020). Recombinant Lactaptin Induces Immunogenic Cell Death and Creates an Antitumor Vaccination Effect in Vivo with Enhancement by an IDO Inhibitor. Molecules.

[111] De Giovanni C., Nanni P., Landuzzi L., Ianzano M.L., Nicoletti G., Croci S., Palladini A., Lollini P.L. (2019). Immune targeting of autocrine IGF2 hampers rhabdomyosarcoma growth and metastasis. BMC Cancer.

[112] Phelps M.P., Yang H., Patel S., Rahman M.M., McFadden G., Chen E. (2018). Oncolytic Virus-Mediated RAS Targeting in Rhabdomyosarcoma. Mol. Ther. Oncolytics.

[113] Merchant M.S., Bernstein D., Amoako M., Baird K., Fleisher T.A., Morre M., Steinberg S.M., Sabatino M., Stroncek D.F., Venkatasan A.M. (2016). Adjuvant Immunotherapy to Improve Outcome in High-Risk Pediatric Sarcomas. Clin. Cancer Res..

[114] Krishnadas D.K., Shusterman S., Bai F., Diller L., Sullivan J.E., Cheerva A.C., George R.E., Lucas K.G. (2015). A phase I trial combining decitabine/dendritic cell vaccine targeting MAGE-A1, MAGE-A3 and NY-ESO-1 for children with relapsed or therapy-refractory neuroblastoma and sarcoma. Cancer Immunol. Immunother..

[115] Hayes C. (2021). Cellular immunotherapies for cancer. Ir. J. Med. Sci..

[116] Lin H., Cheng J., Mu W., Zhou J., Zhu L. (2021). Advances in Universal CAR-T Cell Therapy. Front. Immunol..

[117] Chung H., Jung H., Noh J.Y. (2021). Emerging Approaches for Solid Tumor Treatment Using CAR-T Cell Therapy. Int. J. Mol. Sci..

[118] Thanindratarn P., Dean D.C., Nelson S.D., Hornicek F.J., Duan Z. (2020). Chimeric antigen receptor T (CAR-T) cell immunotherapy for sarcomas: From mechanisms to potential clinical applications. Cancer Treat. Rev..

[119] Kulczycka M., Derlatka K., Tasior J., Lejman M., Zawitkowska J. (2023). CAR T-Cell Therapy in Children with Solid Tumors. J. Clin. Med..

[120] Timpanaro A., Piccand C., Dzhumashev D., Anton-Joseph S., Robbi A., Moser J., Rössler J., Bernasconi M. (2023). CD276-CAR T cells and Dual-CAR T cells targeting CD276/FGFR4 promote rhabdomyosarcoma clearance in orthotopic mouse models. J. Exp. Clin. Cancer Res..

[121] Lake J.A., Woods E., Hoffmeyer E., Schaller K.L., Cruz-Cruz J., Fernandez J., Tufa D., Kooiman B., Hall S.C., Jones D. (2024). Directing B7-H3 chimeric antigen receptor T cell homing through IL-8 induces potent antitumor activity against pediatric sarcoma. J. Immunother. Cancer.

[122] Gattenloehner S., Vincent A., Leuschner I., Tzartos S., Müller-Hermelink H.K., Kirchner T., Marx A. (1998). The fetal form of the acetylcholine receptor distinguishes rhabdomyosarcomas from other childhood tumors. Am. J. Pathol..

[123] Gattenlöhner S., Marx A., Markfort B., Pscherer S., Landmeier S., Juergens H., Müller-Hermelink H.K., Matthews I., Beeson D., Vincent A. (2006). Rhabdomyosarcoma lysis by T cells expressing a human autoantibody-based chimeric receptor targeting the fetal acetylcholine receptor. Cancer Res..

[124] Simon-Keller K., Paschen A., Eichmüller S., Gattenlöhner S., Barth S., Koscielniak E., Leuschner I., Stöbel P., Hombach A., Abken H. (2010). Adoptive T-cell therapy of rhabdomyosarcoma. Pathologe.

[125] Simon-Keller K., Paschen A., Hombach A.A., Ströbel P., Coindre J.M., Eichmüller S.B., Vincent A., Gattenlöhner S., Hoppe F., Leuschner I. (2013). Survivin blockade sensitizes rhabdomyosarcoma cells for lysis by fetal acetylcholine receptor-redirected T cells. Am. J. Pathol..

[126] Huang X., Park H., Greene J., Pao J., Mulvey E., Zhou S.X., Albert C.M., Moy F., Sachdev D., Yee D. (2015). IGF1R- and ROR1-Specific CAR T Cells as a Potential Therapy for High Risk Sarcomas. PLoS ONE.

[127] Xiao W., Wang J., Wen X., Xu B., Que Y., Yu K., Xu L., Zhao J., Pan Q., Zhou P. (2020). Chimeric antigen receptor-modified T-cell therapy for platelet-derived growth factor receptor α-positive rhabdomyosarcoma. Cancer.

[128] Kubo H., Yagyu S., Nakamura K., Yamashima K., Tomida A., Kikuchi K., Iehara T., Nakazawa Y., Hosoi H. (2021). Development of non-viral, ligand-dependent, EPHB4-specific chimeric antigen receptor T cells for treatment of rhabdomyosarcoma. Mol. Ther. Oncolytics.

[129] Shivaprasad N., Xiong Y., Yohe M., Schneider D., Shern J., Baskar S., Dimitrov D., Sorenson P., Orentas R., Khan J. (2016). 649. Developing FGFR4 Chimeric Antigen Receptor CAR T Cell Therapy Against Rhabdomyosarcoma. Mol. Ther..

[130] Alijaj N., Moutel S., Gouveia Z.L., Gray M., Roveri M., Dzhumashev D., Weber F., Meier G., Luciani P., Rössler J.K. (2020). Novel FGFR4-Targeting Single-Domain Antibodies for Multiple Targeted Therapies against Rhabdomyosarcoma. Cancers.

[131] Sullivan P.M., Kumar R., Li W., Hoglund V., Wang L., Zhang Y., Shi M., Beak D., Cheuk A., Jensen M.C. (2022). FGFR4-Targeted Chimeric Antigen Receptors Combined with Anti-Myeloid Polypharmacy Effectively Treat Orthotopic Rhabdomyosarcoma. Mol. Cancer Ther..

[132] Tian M., Wei J.S., Shivaprasad N., Highfill S.L., Gryder B.E., Milewski D., Brown G.T., Moses L., Song H., Wu J.T. (2023). Preclinical development of a chimeric antigen receptor T cell therapy targeting FGFR4 in rhabdomyosarcoma. Cell Rep. Med..

[133] Xiao W., Xu L., Wang J., Yu K., Xu B., Que Y., Zhao J., Pan Q., Gao C., Zhou P. (2024). FGFR4-specific CAR-T cells with inducible caspase-9 suicide gene as an approach to treat rhabdomyosarcoma. Cancer Gene Ther..

[134] Jiang C., Zhao W., Qin M., Jin M., Chang L., Ma X. (2019). CD56-chimeric antigen receptor T-cell therapy for refractory/recurrent rhabdomyosarcoma: A 3.5-year follow-up case report. Medicine.

[135] Shum T., Omer B., Tashiro H., Kruse R.L., Wagner D.L., Parikh K., Yi Z., Sauer T., Liu D., Parihar R. (2017). Constitutive Signaling from an Engineered IL7 Receptor Promotes Durable Tumor Elimination by Tumor-Redirected T Cells. Cancer Discov..

[136] Del Bufalo F., De Angelis B., Caruana I., Del Baldo G., De Ioris M.A., Serra A., Mastronuzzi A., Cefalo M.G., Pagliara D., Amicucci M. (2023). GD2-CART01 for Relapsed or Refractory High-Risk Neuroblastoma. N. Engl. J. Med..

[137] Pezzella M., Quintarelli C., Quadraccia M.C., Sarcinelli A., Manni S., Iaffaldano L., Ottaviani A., Ciccone R., Camera A., D’Amore M.L. (2024). Tumor-derived G-CSF induces an immunosuppressive microenvironment in an osteosarcoma model, reducing response to CAR.GD2 T-cells. J. Hematol. Oncol..

[138] Vivier E., Tomasello E., Baratin M., Walzer T., Ugolini S. (2008). Functions of natural killer cells. Nat. Immunol..

[139] Mechtersheimer G., Staudter M., Majdic O., Dörken B., Moldenhauer G., Möller P. (1990). Expression of HLA-A,B,C, beta 2-microglobulin (beta 2m), HLA-DR, -DP, -DQ and of HLA-D-associated invariant chain (Ii) in soft-tissue tumors. Int. J. Cancer.

[140] Ljunggren H.G., Kärre K. (1990). In search of the ‘missing self’: MHC molecules and NK cell recognition. Immunol. Today.

[141] Cho D., Shook D.R., Shimasaki N., Chang Y.H., Fujisaki H., Campana D. (2010). Cytotoxicity of activated natural killer cells against pediatric solid tumors. Clin. Cancer Res..

[142] Berraondo P., Sanmamed M.F., Ochoa M.C., Etxeberria I., Aznar M.A., Pérez-Gracia J.L., Rodríguez-Ruiz M.E., Ponz-Sarvise M., Castañón E., Melero I. (2019). Cytokines in clinical cancer immunotherapy. Br. J. Cancer.

[143] Boerman G.H., van Ostaijen-ten Dam M.M., Kraal K.C., Santos S.J., Ball L.M., Lankester A.C., Schilham M.W., Egeler R.M., van Tol M.J. (2015). Role of NKG2D, DNAM-1 and natural cytotoxicity receptors in cytotoxicity toward rhabdomyosarcoma cell lines mediated by resting and IL-15-activated human natural killer cells. Cancer Immunol. Immunother..

[144] Wagner J., Pfannenstiel V., Waldmann A., Bergs J.W.J., Brill B., Huenecke S., Klingebiel T., Rödel F., Buchholz C.J., Wels W.S. (2017). A Two-Phase Expansion Protocol Combining Interleukin (IL)-15 and IL-21 Improves Natural Killer Cell Proliferation and Cytotoxicity against Rhabdomyosarcoma. Front. Immunol..

[145] Rademacher M.J., Cruz A., Faber M., Oldham R.A.A., Wang D., Medin J.A., Schloemer N.J. (2021). Sarcoma IL-12 overexpression facilitates NK cell immunomodulation. Sci. Rep..

[146] Gassmann H., Thiede M., Weiss J., Biele E., Flohe L., Lachermaier H., Prexler C., Evdokimova V., Radvanyi L., Akhtar I. (2024). Cytokine screening identifies TNF to potentially enhance immunogenicity of pediatric sarcomas. Front. Immunol..

[147] Vela M., Bueno D., González-Navarro P., Brito A., Fernández L., Escudero A., Valentín J., Mestre-Durán C., Arranz-Álvarez M., Pérez de Diego R. (2019). Anti-CXCR4 Antibody Combined With Activated and Expanded Natural Killer Cells for Sarcoma Immunotherapy. Front. Immunol..

[148] Lang P., Pfeiffer M., Müller I., Schumm M., Ebinger M., Koscielniak E., Feuchtinger T., Föll J., Martin D., Handgretinger R. (2006). Haploidentical stem cell transplantation in patients with pediatric solid tumors: Preliminary results of a pilot study and analysis of graft versus tumor effects. Klin. Padiatr..

[149] Llosa N.J., Cooke K.R., Chen A.R., Gamper C.J., Klein O.R., Zambidis E.T., Luber B., Rosner G., Siegel N., Holuba M.J. (2017). Reduced-Intensity Haploidentical Bone Marrow Transplantation with Post-Transplant Cyclophosphamide for Solid Tumors in Pediatric and Young Adult Patients. Biol. Blood Marrow Transplant..

[150] Pérez-Martínez A., Leung W., Muñoz E., Iyengar R., Ramírez M., Vicario J.L., Lassaletta A., Sevilla J., González-Vicent M., Madero L. (2009). KIR-HLA receptor-ligand mismatch associated with a graft-versus-tumor effect in haploidentical stem cell transplantation for pediatric metastatic solid tumors. Pediatr. Blood Cancer.

[151] Marofi F., Abdul-Rasheed O.F., Rahman H.S., Budi H.S., Jalil A.T., Yumashev A.V., Hassanzadeh A., Yazdanifar M., Motavalli R., Chartrand M.S. (2021). CAR-NK cell in cancer immunotherapy; A promising frontier. Cancer Sci..

[152] Imai C., Iwamoto S., Campana D. (2005). Genetic modification of primary natural killer cells overcomes inhibitory signals and induces specific killing of leukemic cells. Blood.

[153] Gossel L.D.H., Heim C., Pfeffermann L.M., Moser L.M., Bönig H.B., Klingebiel T.E., Bader P., Wels W.S., Merker M., Rettinger E. (2021). Retargeting of NK-92 Cells against High-Risk Rhabdomyosarcomas by Means of an ERBB2 (HER2/Neu)-Specific Chimeric Antigen Receptor. Cancers.

[154] Lam P.Y., Omer N., Wong J.K.M., Tu C., Alim L., Rossi G.R., Victorova M., Tompkins H., Lin C.Y., Mehdi A.M. (2025). Enhancement of anti-sarcoma immunity by NK cells engineered with mRNA for expression of a EphA2-targeted CAR. Clin. Transl. Med..

[155] Heim C., Hartig L., Weinelt N., Moser L.M., Salzmann-Manrique E., Merker M., Wels W.S., Tonn T., Bader P., Klusmann J.H. (2024). Bortezomib promotes the TRAIL-mediated killing of resistant rhabdomyosarcoma by ErbB2/Her2-targeted CAR-NK-92 cells via DR5 upregulation. Mol. Ther. Oncol..

[156] Schmidt-Wolf I.G., Negrin R.S., Kiem H.P., Blume K.G., Weissman I.L. (1991). Use of a SCID mouse/human lymphoma model to evaluate cytokine-induced killer cells with potent antitumor cell activity. J. Exp. Med..

[157] Leuci V., Donini C., Grignani G., Rotolo R., Mesiano G., Fiorino E., Gammaitoni L., D’Ambrosio L., Merlini A., Landoni E. (2020). CSPG4-Specific CAR.CIK Lymphocytes as a Novel Therapy for the Treatment of Multiple Soft-Tissue Sarcoma Histotypes. Clin. Cancer Res..

[158] Merker M., Pfirrmann V., Oelsner S., Fulda S., Klingebiel T., Wels W.S., Bader P., Rettinger E. (2017). Generation and characterization of ErbB2-CAR-engineered cytokine-induced killer cells for the treatment of high-risk soft tissue sarcoma in children. Oncotarget.

[159] Merker M., Wagner J., Kreyenberg H., Heim C., Moser L.M., Wels W.S., Bonig H., Ivics Z., Ullrich E., Klingebiel T. (2020). ERBB2-CAR-Engineered Cytokine-Induced Killer Cells Exhibit Both CAR-Mediated and Innate Immunity Against High-Risk Rhabdomyosarcoma. Front. Immunol..

[160] Moser L.M., Heim C., Koschade S.E., Wendel P., Bozkurt S., Harenkamp S., Kreyenberg H., Merker M., Munch C., Gradhand E. (2025). CAR-CIK vs. CAR-T: Benchmarking novel cytokine-induced killer cells as solid tumor immunotherapy in ErbB2^+^ rhabdomyosarcoma. Front. Immunol..

[161] Gupta A., Cripe T.P. (2022). Immunotherapies for Pediatric Solid Tumors: A Targeted Update. Paediatr. Drugs.

[162] Wedekind M.F., Denton N.L., Chen C.Y., Cripe T.P. (2018). Pediatric Cancer Immunotherapy: Opportunities and Challenges. Paediatr. Drugs.

[163] Babu S., Krishnan M. (2024). Catalysts of change: Immunotherapy’s frontier in oral oncology. Oral Oncol. Rep..

[164] Wood G.E., Meyer C., Petitprez F., D’Angelo S.P. (2024). Immunotherapy in Sarcoma: Current Data and Promising Strategies. American Society of Clinical Oncology Educational Book.

[165] Pilavaki P., Panagi M., Arifi S., Jones R.L., Stylianopoulos T., Constantinidou A. (2022). Exploring the landscape of immunotherapy approaches in sarcomas. Front. Oncol..

[166] Peng L., Sferruzza G., Yang L., Zhou L., Chen S. (2024). CAR-T and CAR-NK as cellular cancer immunotherapy for solid tumors. Cell. Mol. Immunol..

[167] Ortiz M.V., Bender J.L.G. (2024). Delays in Pediatric Evaluation of New and Relevant Cancer Therapies. J. Pediatr..

[168] Food and Drug Administration Cancer Clinical Trial Eligibility Criteria: Minimum Age Considerations for Inclusion of Pediatric Patients. Guidance for Industry and IRBs. https://www.fda.gov/regulatory-information/search-fda-guidance-documents/cancer-clinical-trial-eligibility-criteria-minimum-age-considerations-inclusion-pediatric-patients.

[169] Yan A.P., Venkatramani R., Bradley J.A., Lautz T.B., Urla C.I., Merks J.H.M., Oberoi S. (2023). Clinical Characteristics, Treatment Considerations, and Outcomes of Infants with Rhabdomyosarcoma. Cancers.

[170] Makimoto A. (2022). Optimizing Rhabdomyosarcoma Treatment in Adolescents and Young Adults. Cancers.

[171] Vo K.T., Parsons D.W., Seibel N.L. (2020). Precision Medicine in Pediatric Oncology. Surg. Oncol. Clin. N. Am..

[172] Heipertz A.E., Pajtler K.W., Pfaff E., Schramm K., Blattner-Johnson M., Milde T., Jones B.C., Zuliani C., Hutter C., Lohi O. (2023). Outcome of Children and Adolescents With Relapsed/Refractory/Progressive Malignancies Treated With Molecularly Informed Targeted Drugs in the Pediatric Precision Oncology Registry INFORM. JCO Precis. Oncol..

[173] McCabe M.G., Geoerger B., Chesler L., Hargrave D., Parsons D.W., van Tilburg C.M., Schleiermacher G., Hickman J.A., George S.L. (2024). Precision Medicine for Childhood Cancer: Current Limitations and Future Perspectives. JCO Precis. Oncol..

